# Comparative Study of Three Dyes’ Adsorption onto Activated Carbon from *Chenopodium quinoa* Willd and *Quillaja saponaria*

**DOI:** 10.3390/ma15144898

**Published:** 2022-07-14

**Authors:** Diana Abril, Victor Ferrer, Yaneris Mirabal-Gallardo, Gustavo Cabrera-Barjas, Cristina Segura, Adolfo Marican, Alfredo Pereira, Esteban F. Durán-Lara, Oscar Valdés

**Affiliations:** 1 Departamento de Biología y Química, Facultad de Ciencias Básicas, Universidad Católica del Maule, Talca 3460000, Chile; dabril@ucm.cl; 2 Unidad de Desarrollo Tecnológico, UDT, Universidad de Concepción, Av. Cordillera 2634, Parque Industrial Coronel, Coronel 4190000, Chile; v.ferrer@udt.cl (V.F.); g.cabrera@udt.cl (G.C.-B.); c.segura@udt.cl (C.S.); 3 Centro Nacional de Excelencia para la Industria de la Madera (CENAMAD), Pontificia Universidad Católica de Chile, Av. Vicuña Mackena 4860, Santiago 7820436, Chile; 4 Instituto de Ciencias Químicas Aplicadas, Facultad de Ingeniería Civil, Universidad Autónoma de Chile, Sede Talca, Talca 3460000, Chile; yaneris.mirabal01@uautonoma.cl; 5 Instituto de Química de Recursos Naturales, Universidad de Talca, Talca 3460000, Chile; amarican@utalca.cl (A.M.); alpereira@utalca.cl (A.P.); 6 Bio & NanoMaterials Laboratory, Drug Delivery and Controlled Release, Departamento de Microbiología, Facultad de Ciencias de la Salud, Universidad de Talca, Talca 3460000, Chile; eduran@utalca.cl; 7 Centro de Investigación de Estudios Avanzados del Maule (CIEAM), Vicerrectoría de Investigación y Postgrado, Universidad Católica del Maule, Talca 3460000, Chile

**Keywords:** crystal violet dye, sunset yellow FCF dye, tartrazine dye, adsorption, dye removal, activated carbon

## Abstract

The present study shows porous activated carbon obtained from *Chenopodium quinoa* Willd and *Quillaja saponaria* and their use as potential adsorbents to remove three types of dyes from aqueous solutions. The adsorption results were compared with commercial charcoal to check their efficiency. All porous carbon materials were activated using carbon dioxide and steam and fully characterized. Moreover, the steam-activated samples exhibited a high total pore volume with a BET surface area of around 800 m^2^ g^−1^. Batch adsorption experiments showed that commercial charcoal is the charcoal that offered the best adsorption efficiency for tartrazine and sunset yellow FCF. However, in the case of crystal violet, all activated carbons obtained from *Chenopodium quinoa* Willd and *Quillaja saponaria* showed the best captures, outperforming commercial charcoal. Molecular dockings of the dyes on the commercial charcoal surface were performed using AutoDock Vina. The kinetic results of the three isotherm’s models for the present data follow the order: Langmuir~Freundlich > Temkin.

## 1. Introduction

During recent years, dyes and pigments in the textile, leather, plastic, food processing, cosmetics, paper, printing, pharmaceutical, and dye manufacturing industries have increased exponentially. Currently, approximately 2 × 10^5^ tons of natural and synthetic dyes are released into the environment yearly, which is responsible for river and spring pollution [1]. Different types of dyes are present in the market, and they can be classified into three types based on their nuclear structures: anionic, nonionic, and cationic [2]. Therefore, in this research, we used two organic anionic dyes, namely tartrazine (TAR) and sunset yellow FCF (SSY), and crystal violet (CV), a cationic dye. It is essential to mention that in literature reports, cationic dyes have been regarded as more toxic than anionic ones [3].

TAR and SSY are synthetic anionic dyes used commercially at very low concentrations as food additives in the pharmaceutical and cosmetics industries [4]. On the other hand, CV is extensively used in medicine as a biological stain, for identifying bloody fingerprints as a protein dye, and in various commercial textile operations [5]. The high concentrations of these dyes are very harmful to human health, producing infertility, thyroid cancer, asthma, migraines, eczema, lupus, and hyperactivity [6,7]. Furthermore, these molecules are non-biodegradable and can survive in various environments. Therefore, it is vital to reduce or eliminate the concentrations of these dyes from effluents before discharging them into water bodies. Different methods can be adopted and applied to mitigate this environmental problem, such as microbial and biological treatment, photo-catalytic reduction, chemical oxidation, ion exchange, membrane osmosis, and adsorption [8]. However, the methods described above suffer from one or more limitations (efficiency, cost-effectiveness, availability, management, etc.) and have proven unreliable for industrial applications, except for the adsorption method.

The adsorption technique has received significant attention due to being the most helpful, inexpensive, reliable, and simple method for removing dyes. However, the effectiveness of the adsorption process for dye removal depends on the adsorbent used, with activated carbon being the most commonly used material. Specifically, developing activated carbons using vegetable sources could be an alternative to producing environmentally friendly, low-cost adsorbents to remove dyes from solutions.

Activated carbon (AC) is a well-known material that separates and removes unwanted substances in gas or liquid industrial effluents. The raw materials widely used initially for producing commercial activated carbon are coal, bone char, peat, petroleum coke, lignite, wood, and other biomass sources [9]. AC has many characteristics that favor it, such as its high porosity, surface area, and adsorption capacity [10,11]. AC can be made from any low-cost material that is high in carbon and low in inorganic matter [12,13]. For this reason, in recent years, research has focused on using alternative raw materials for making AC, such as low-hard woods (e.g., pine), mineral coals, vegetable shells, and agricultural residues. This last material is of great interest given its availability, low cost, and renewable nature. For the removal of dye, sorbents based on coal have been employed successfully [14,15], but occasionally its application is constrained by activated carbon’s expensive price [16]. In this way, activated carbons made from agro-industrial waste such as rice husk [17], pecan nut shells [18], and cherry stones [19] have been used to remove crystal violet (65 mg/g), tartrazine (46 mg/g), and sunset yellow (125 mg/g) dyes with the same or even better results than those used commercially.

The quinoa (*Chenopodium quinoa* Willd) plant is a pseudocereal highly consumed in South America since ancestral times by local people. Due to its high protein quality and mineral content, this crop has been widely cultivated in other continents, becoming a new producer [20,21]. As a result of industrial processing of the grain, tons of quinoa husk byproducts are obtained and must be disposed of in landfills. Husk biomass contains proteins (13.5 wt%), saponin compounds (<5 wt%) with biological activity, and lignocellulosic compounds in high concentrations (>85 wt%) [22,23,24,25]. So far, any sizeable industrial use of this waste biomass is unknown, even when some approach to isolating saponins and using the husk in cosmetics has been made on a low scale. On the other hand, Chile’s commercial exploitation of quillay (*Quillaja saponaria*) tree bark is well established [26]. The tree was used to obtain saponin-rich extracts with surfactant properties, which have applications in medicine, pharma, agriculture, cosmetics, and food industries [27]. After extraction, the residual lignocellulosic biomass must be disposed of, and in some cases, it is pelletized and used in cattle feed. Therefore, this is a limited and low-value application for this abundant byproduct. Thus, there is an opportunity to obtain high-value products from agro-forest wastes derived from industrial processes. Moreover, societies must develop sustainable strategies that can be part of the circular economy trend being promoted today.

One method used to prepare AC is the two-step physical activation procedure. In this method, the precursor is carbonized first under an inert atmosphere. Then, the resulting char is subjected to partial and controlled gasification at a high temperature with steam, carbon dioxide, air, or a mixture [28]. However, carrying out this activation in one step (carbonization-activation) is desirable due to lower energy consumption, capital expenditure, and processing time, which can significantly improve the process economics [29]. In addition, steam and CO_2_ as activating agents are an advantage because they are less expensive and less corrosive than the chemical reagents used in the chemical activation method (KOH, H_3_PO_4_, ZnCl_2_) [30].

To our knowledge, few investigations have been related to removing the dyes mentioned above, using quillay and quinoa residues as a precursor of activated carbon. In this work, physical activation using CO_2_ and steam as the activating agents was employed to prepare the activated carbons. These samples were tested by removing the solution’s crystal violet, tartrazine, and sunset yellow FCF dyes. The activated carbons were characterized by Fourier transform infrared spectroscopy (FT-IR), X-ray diffraction (XRD), nitrogen and ammonia sorption methods, and scanning electron microscopy (SEM). In addition, computational methods are incorporated to simulate the adsorption of dyes on activated carbons, as well as studies of adsorption kinetics and equilibrium.

## 2. Materials and Methods

### 2.1. Chemicals and Reagents

**Figure 1:**
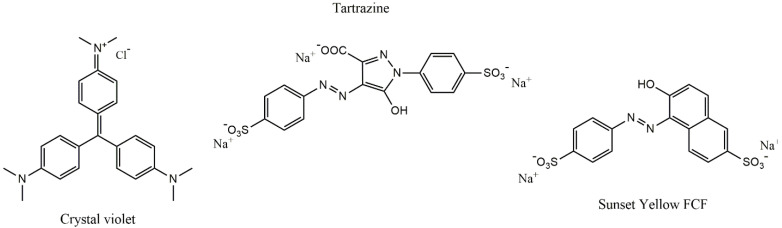
The molecular structures of dyes.

Crystal violet ([4-[bis[4-(dimethylamino)phenyl]methylidene]cyclohexa-2,5-dien-1-ylidene]-dimethylazanium;chloride), tartrazine (trisodium1-(4-sulfonatophenyl)-4-(4-sulfonatophenylazo)-5-pyrazolone-3-carboxylate), sunset yellow FCF (Disodium 6-hydroxy-5-[(4-sulfophenyl)azo]-2-naphthalenesulfonate), and activated charcoal (AC, Norit GAC 1240, S_BET_ = 947 m^2^/g) were purchased from Sigma-Aldrich (St. Louis, MO, USA), and used without purification. The chemical structures of the dyes are shown in Figure 1.

### 2.2. Activated Carbons Preparation

**Figure 2:**
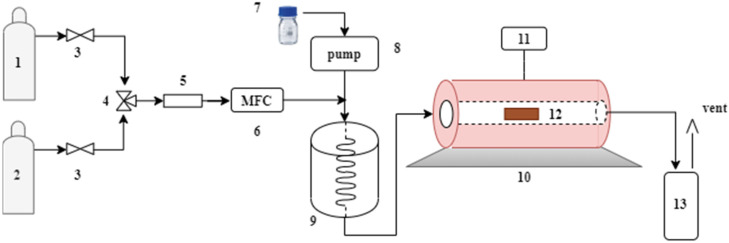
Schematic diagram of the experimental setup. (**1**) N_2_. (**2**) CO_2_. (**3**) On-off valve. (**4**) 3-way valve. (**5**) Filter. (**6**) Mass flow controller. (**7**) Water. (**8**) Peristaltic pump. (**9**) Steam generator. (**10**) Oven. (**11**) Temperature controller. (**12**) Sample. (**13**) Condenser.

Physical activation in one step was done employing CO_2_ (Linde, 99.5%) and steam as activating agents. The experimental apparatus used in this study for the activated carbons obtained from quillay (*Quillaja saponaria*, QS) tree bark biomass and quinoa husk (*Chenopodium quinoa* Willd., variety Regalona, CQW) is shown in Figure 2. In the CO_2_ activation, the QS raw materials (10 g) were treated at 800 °C for two hours with a CO_2_ flow rate of 100 mL/min and a heating rate of 10 °C/min. For the CQW sample, the activation was done at 750 °C for one hour, keeping the other parameters at the same values. The resulting samples were designated as QS-CO_2_ and CQW-CO_2_, respectively.

The raw material was activated at 850 °C for one hour for the steam activation, keeping a steam/N_2_ ratio equal to 4. Next, the samples were heated up to the activation temperature in N_2_ flow (134 mL/min) with a heating rate of 10 °C/min. Once the temperature was reached, the steam was introduced into the furnace. The steam was generated by injecting 0.4 mL/min of liquid water into an evaporator by a peristaltic pump (equivalent to 534 mL/min of steam determined at 20 °C and 101.32 kPa). After activation, the samples designated as QS-H_2_O and CQW-H_2_O were cooled to room temperature under N_2_ flow.

For comparison, the raw material was only submitted to carbonization (Pyrolysis) in the horizontal furnace at 500 °C for two hours with a heating rate of 10 °C/min and an N_2_ (Linde, 99.995%) flow rate of 150 mL/min. Therefore, these samples were referred to as QS-P, and CQW-P, respectively.

The resultant activated carbons were weighed to determine their yield. Here, the yield (*Y*) of pyrolytic char was defined as the ratio of the sample weight after pyrolysis or activation to the weight of the raw material according to the following equation:

(1) $$Y\left(\%\right)=\frac{W_{f}}{W_{i}}*100$$

where *W_f_* and *W_i_* are the mass of the resulting char and the initial mass of the raw olive stone, respectively.

### 2.3. Characterization of Activated Carbon

The characterization of all activated carbons prepared from QS and CQW was conducted using Fourier transform infrared spectroscopy (FT-IR), X-ray diffraction (XRD), nitrogen, ammonia sorption methods, and scanning electron microscopy (SEM). FT-IR analysis of all the activated carbon prepared was carried out using a Shimadzu/FT-IR-8400S spectrometer with KBr pellets from 500 to 4000 cm^−1^. The activated carbons with and without dyes were observed using scanning electron microscopy. The surface analysis was carried out on a JEOL JSM-6500F field emission SEM at 15 kV. Wavelength dispersive X-ray fluorescence (WDXRF) and X-ray diffraction (XRD) analysis of activated carbons were conducted according to standard test methods of ASTM E1621-21, using a fluorescence spectrometer of dispersive wavelength of 1 kW brand BRUKER model S8 TIGER. The X-ray source was a rhodium (Rh) tube [31].

The nitrogen and ammonia sorption isotherms were used to characterize the surface area, micropore volume, and adsorptive capacity using a Micromeritics 3Flex adsorption analyzer. A 50 mg sample was treated in He flow at 300 °C for 4 h before measurement. The Brunauer–Emmet–Teller (BET) method was used to determine the specific surface area. Total pore volume was calculated at a pressure where all pores were filled with nitrogen gas, approximately at a P/P_0_ of 0.999. The micropore volume was calculated using the Dubinin–Radushkevich equation [32,33]. For NH_3_ adsorption, the treated sample was exposed to the increasing pressure of anhydrous NH_3_ (Indura, 99.5%) up to 78 kPa at room temperature. The evaluation of ammonia adsorption is interesting because it is both industrially important and a major pollutant that must be removed from many industrial gas streams.

Other parameters such as pH, iodine numbers, proximate, and ultimate analysis were also done. The pH parameter was measured in a filtrate suspension of AC with a concentration of 2 g/L in distilled water for 6 h. Proximate analysis was done following the ASTM D1506 and ISO 562 standards to determine the content of ash and volatiles, respectively, while the moisture was determined after drying the sample at 105 °C overnight. Ultimate analysis was performed on a Leco CHNS 628 elemental analyzer. Finally, the iodine number (milligrams of iodine adsorbed by 1 g of carbon) was estimated by mixing the powder samples of activated carbons with a 0.1 N iodine solution shaken occasionally and then titrating the solution against a standardized 0.1 N Na_2_S_2_O_3_ solution [34].

### 2.4. Batch Adsorption Studies

Before batch adsorption experiments, TAR, CV, and SSY stock solutions (1000 mg/L) were used as dye models and were prepared using double distilled water. Dyes of commercial purity were used without further purification. All solutions used in the experiments were prepared by diluting the stock solution to predetermined concentrations. Batch adsorption experiments were carried out to investigate the adsorption performance of the carbon samples, following the procedure described by Ahmad with slight modifications [7]. It is essential to mention that the adsorption of TAR, CV, and SSY dyes was studied separately, using the same conditions for all experiments. The experimental run was conducted using 30 mg of activated carbon per experiment in 10 mL amber vials at room temperature. The adsorption equilibrium was investigated for three different dye concentrations: aqueous solutions of 100, 250, and 400 mg/L. The mixtures were shaken at 120 rpm using an orbital shaker (ALLSHENG Model OS-200/PP1). In addition, the adsorbent adsorbate contact time varied between 30 and 240 min. Finally, to quantify the remaining dye content, the supernatant was separated by filtration using Whatman paper (N° 42) and quantified by a UV/Vis spectrophotometer (Shimadzu UV–Vis 160 A) at the wavelength of 426, 543, and 481 nm for TAR, CV, and SSY, respectively. The following equation calculates the percent removal of dye from the solution:

(2) $$\%\;removal=\frac{C_{0}-C_{f}}{C_{0}}*100$$

where *C*_0_ and *C_f_* (mg/L) are the dye solution concentrations initially and finally, respectively. In addition, the adsorption capacity *q_e_* (mg/g) after equilibrium was calculated by the mass balance relationship equation as follows:

(3) $$q_{e}=\frac{\left(C_{0}-C_{f}\right)V}{W}$$

where *V* (L) is the volume of the solution and *W* (g) is the mass of adsorbate.

An experimental design based on 22 tests was used to evaluate the effects of selected independent variables such as contact time and the adsorption of dyes over adsorbents on the responses. We considered contact times between active carbon and dye solutions of 20 and 120 min, whereas the concentration values of the dye solutions were described before. The values of the experimental variables were coded between −1 and 1, considering the same statistical weight. It is essential to mention that this design helps optimize the sorption process using a small number of experimental runs. Finally, the used mass of adsorbents was approximately 0.03 g in all experiments. The experimental design is presented in Table 1.

### 2.5. Computational Methods

The CV, TAR, SSY, and AC surface structures were designed and built through MarvinSketch software v21.9, ChemAxon (https://www.chemaxon.com, accessed on 22 May 2020). Geometries of each structure were optimized by the lowest energy conformer search using the MMFF94 force field [35]. Molecular dockings of CV, TAR, and SSY on the AC surface were performed using AutoDock Vina in PyRx v0.8 software [36,37]. Structures were loaded and then transformed to PDBQT. The AC conformation was considered a rigid unit, while the dyes were allowed to be flexible and adaptable to the target. A grid box was set to cover the AC surface with a size of x = 25 Å, y = 25 Å, and z = 25 Å, and an origin point at x = −11.4, y = −1.3, and z = 1.1 with the default exhaustiveness value of 8. Using BIOVIA Discovery Studio Visualizer v21 [38], the docked complexes with the lowest binding affinity values were further analyzed for intermolecular interaction.

### 2.6. Isotherm Models and Kinetic Study

Three isotherms models, namely Langmuir, Temkin, and Freundlich, were employed to study the adsorption data [39]. The Langmuir, Freundlich, and Temkin equations are as follows:

(4) $$\mathrm{Langmuir}\;\mathrm{equation}:\;\frac{1}{q_{e}}=\frac{1}{q_{max}K_{L}C_{e}}+\frac{1}{q_{max}}$$

(5) $$\mathrm{Freundlich}\;\mathrm{equation}:\;Logq_{e}=LogK_{F}+\frac{1}{n}LogC_{e}$$

(6) $$\mathrm{Temkin}\;\mathrm{equation}:\;q_{e}=a+bLnC_{e}$$

where *q_e_* is the amount adsorbed at equilibrium (mg/g) and *C_e_* is the equilibrium dye concentration in the solution (mg/L). Other parameters such as *q_max_* (mg/g), *K_L_*, *n*, and *K_F_*, which represent adsorption capacity under the experimental conditions, constant related to the energy of adsorption, a parameter indicative of bond energies between dye ion and the adsorbent, and the continuous corresponding to bond strength, respectively. It is important to note that these last-mentioned parameters can be determined by linear regression of the experimental data, applying the Langmuir and Freundlich equations.

For the case of the Langmuir isotherm, its adsorption characteristics can be estimated by the equilibrium parameter (*R_L_*) [40]:

(7) $$R_{L}=\frac{1}{1+K_{L}C_{0}}$$

where *C*_0_ corresponds to the highest initial dye concentration. The *R_L_* value indicates if the adsorption is unfavorable (*R_L_* > 1), favorable (0 < *R_L_* < 1), linear (*R_L_* = 1), or reversible (*R_L_* = 0).

The experimental data were fitted to the pseudo-first and second-order adsorption kinetic models. These models are widely used to predict adsorption processes. The pseudo-first-order kinetic model is expressed as [41]:

(8) $$q_{t}=q_{e,c}\left(1-e^{-k_{1}t}\right)$$

The following equation expressed the pseudo-second-order rate model [35]:

(9) $$q_{t}=\frac{k_{2}q_{e,c}{}^{2}t}{1+k_{2}q_{e,c}{}^{2}t}$$

where *q_e,c_* represents the calculated amount of dye adsorbed at the equilibrium (mg/g), *k*_1_ is the pseudo-first-order rate constant (h^−1^), *q_t_* is the amount of dye adsorbed at any time (mg/g), and *k*_2_ is the pseudo-second-order rate constant (mg/(g h)). Non-linear regression analysis was used to estimate the parameters *q_e,c_*, *k*_1_, and *k*_2_. The degree of fit was evaluated from the correlation (R^2^) and Chi-square (χ^2^) coefficients.

## 3. Results

### 3.1. Physicochemical Characterization of Adsorbents

Following the procedure described above, the pyrolyzed and activated carbon from QS and CQW were produced and characterized. In addition, carbon dioxide and steam were used as activating agents since both activated techniques are frequently used to produce activated carbon.

The proximate and ultimate analysis of the activated carbons is given in Table 2. The raw materials had a high content of volatile matter and low fixed carbon, especially for the CQW sample. Furthermore, this last sample registered a high ash content (15.19%) concentrated in the char with the pyrolysis and activation process.

The proximate analysis data showed a general progression of fixed carbon increase from the raw material to the char and finally to the activated carbon. In contrast, their volatile content showed a reversed trend, as expected. The conversion of volatile matter into gaseous products increased with pyrolysis at 500 °C and activation above 800 °C. Increasing devolatilization steadily increased the char’s fixed carbon and ash contents [42]. Ultimate analysis reported values similar to other biomass and commercial activated carbons [43,44]. An important content of C was registered in raw materials, making them a good precursor for activated carbon. The H percentage decreased after pyrolysis and activation treatments, remaining low in the nitrogen content, varying between 0.4–1.89%.

**Figure 3:**
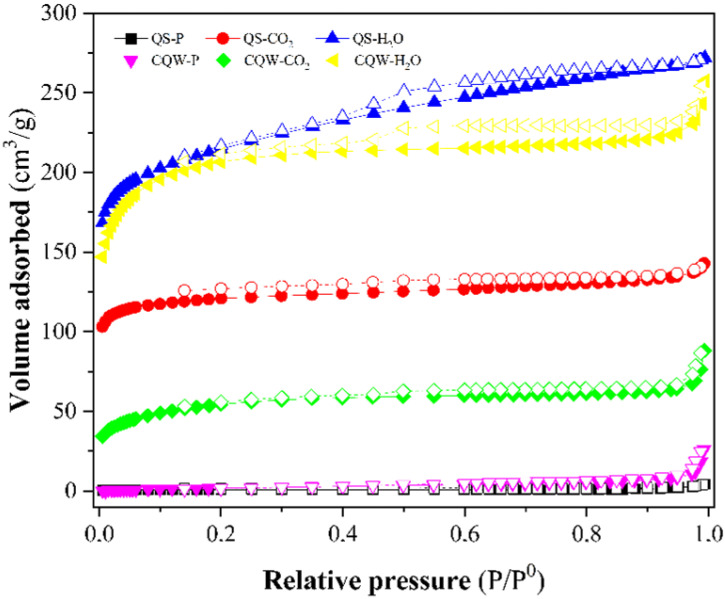
N_2_ adsorption-desorption isotherms curves at 196 °C of samples. Closed symbols: N_2_ adsorption curve and open symbols: N_2_ desorption curve.

Figure 3 depicts the N_2_ adsorption-desorption isotherms of the pyrolytic samples and physically activated carbons. The isotherms for activated carbons were typical Type I microporous carbons (according to IUPAC classification [45]), although a mesoporosity contribution could be noticed. According to the IUPAC nomenclature, the isotherms also showed a type H4 hysteresis loop (according to IUPAC classification [45]) due to capillary condensation during the adsorption-desorption process that corresponds to a bimodal micro-mesoporous structure and is associated with narrow slit-like pores [46].

Table 3 reports the BET surface area of samples. CO_2_ and steam activations were effective for generating porosity and increasing the surface area, especially for steam-activated samples with the highest surface area (~800 m^2^ g^−1^) and total pore volume (V_tot_). These high surface area values are comparable with those reported for the commercial AC [47]. However, the pyrolysis of the starting materials did not lead to the development of char porosity, registering the lowest surface area and the highest average pore diameter for QS-P and CQW-P samples.

In general, we obtained a yield below 28%. During the pyrolysis process, weight loss occurred due to the evolution of volatile material. In the case of activation with CO_2_ and steam, the yield was lower than in pyrolytic samples. This behavior was due to the other reaction that occurred during the pyrolysis [48]:

$$C+CO_{2}\to CO$$

$$C+H_{2}O\to \mathrm{CO}+H_{2}$$

It was noticed that low yield (high burn-off) promoted the high surface area. An increase in the surface area with the burn-off in activated carbons prepared by CO_2_ and steam activation using corn cob as raw material has been reported by Chang et al. [28]. These authors concluded that gasification processes using CO_2_ and steam remove disorganized materials, develop microporosity, and widen the micropore with increasing burn-off.

The pH variation among prepared samples may be explained by differences in their ash content. Carbons with a pH of ~8 (QS-P and QS-CO_2_) had relatively low ash contents, while those around 10–11 had high ash contents. It has been reported that the pH of commercial carbons is due to inorganic constituents originating from the precursor or added during manufacture. Therefore, the samples’ high ash content may explain their mineral richness, which may have contributed to their relatively high pHs [49].

The iodine number was used to monitor the development of the micro-porosities of the prepared samples. It is known that this parameter can be used as an approximation for the surface area and microporosity of active carbons with good precision [50]. The carbons obtained by steam activation registered the highest iodine number (Table 3), indicating that this activation led to the development of a highly porous structure and was correlated with the highest total pore volume obtained. The other carbons showed low iodine numbers, which could be related to creating meso and macropore structures [51]. Other researchers [51,52,53] have reported similar iodine number values for carbons derived from biomass.

**Figure 4:**
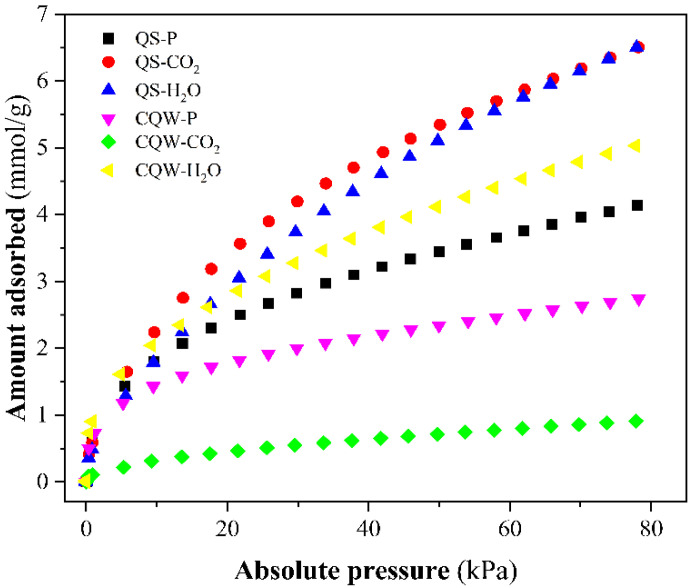
NH_3_ adsorption isotherms at 20 °C of the prepared samples.

On the other hand, NH_3_ adsorption isotherms of samples at 20 °C and gas pressure up to 78 kPa were plotted in Figure 4. From Figure 4, we determined the maximum amount of NH_3_ adsorbed, reported in Table 3. The best NH_3_ adsorption capacity was reported for QS-H_2_O. The values obtained are similar to those reported in the literature [54,55,56], although slightly lower than other carbons [54,57,58]. The pH values registered for most carbons (Table 3) indicated a basic surface. It could explain the low capacity to adsorb the basic molecule of ammonia-based on the acid-base interaction (or pH effect) [59].

### 3.2. Adsorption of Dyes

All activated and pyrolyzed carbons obtained from QS and CQW were evaluated for their dye adsorption capacity in an aqueous solution following the above procedure. In addition, these results were compared with those obtained by commercial charcoal (AC) to test its effectiveness and possible industrial use. Table 4 compares the capture capabilities of the different types of prepared samples for dye solutions. From Table 4, in the case of TAR and SSY, AC is the charcoal that showed the best adsorption efficiency, followed by CQW-P, QS-H_2_O, CQW-CO_2_, and QS-H_2_O, respectively. However, a different behavior was revealed in the case of CV. In this case, all activated carbons obtained from CQW and QS offered the best captures, followed by far outperforming AC. This behavior was mainly caused by the possible negative surface charge, enhancing the electrostatic interaction between the CQW charcoals and the positively charged CV molecules. This probable statement was in agreement with the results obtained by the analysis of the docking-based interactions discussed in the next section.

Concerning the adsorption of each dye on the prepared carbons, a higher adsorption capacity was demonstrated for the CV. In this case, for steam-activated quinoa carbon (CQW-H_2_O), a value of 12.67 mg L dye/mg charcoal (126.7 mg/g) was obtained, closely followed by the same sample activated with CO_2_ (CQW-CO_2_ = 11.77 mg L dye/mg charcoal, 117.7 mg/g) and quillay activated with steam (QS-H_2_O = 11.21 mg L dye/mg charcoal, 112.1 mg/g). The quillay sample activated with CO_2_ (QS-CO_2_) produced the lowest adsorption capacity of all the dyes tested. This high adsorption capacity of the CV dye may be due to the affinity of the negatively charged carbon surface with the cationic dye, as mentioned previously [60]. Additionally, the high pH of the carbon (see Table 3) could influence the adsorption process because different ionic species and surface electrical charges arise when there is a pH charge [61,62]. A high electrostatic interaction exists between the negatively charged surface of activated carbon and cationic dye molecules at high pH levels, which promotes maximum dye biosorption. Foroutan et al. carried out CV adsorption tests on lemon wood carbons, concluding that the adsorption efficiency of this dye increased in an alkaline medium [63].

In the case of anionic dyes, lower adsorption of these was recorded compared to the cationic dye because cellulosic materials in aqueous conditions have a negative charge, creating a repulsive force between the adsorbate and the adsorbent surface [60]. It has been reported that TAR adsorption is favored at an acidic pH (pH = 2.5) [64]. Because the adsorbent surface is positively charged, there will be a stronger electrostatic interaction between positively charged adsorbent particles and negatively charged adsorbate species, which will result in more tartrazine dye being absorbed. This same behavior was reported for SSY dye adsorbed onto activated carbon derived from cassava sievate [4].

The TAR adsorption capacity obtained for the samples is higher than those reported in the literature for soil (83 mg/g) [65], plant-derived activated carbon (46 mg/g) [18], and cassava sievate biomass (21 mg/g) [4]. For SSY, the adsorption capacity was similar to that obtained for *Rhizopus arrhizus* biomass (60 mg/g) [66], and Ag nanoparticle-loaded AC (37 mg/g) [67], but lower than amberlite (131 mg/g) [68] and alligator weed-activated carbon (132 mg/g) [69].

Although the activation methods promoted an increase in the surface area, no significant effect of this parameter on the adsorptive capacity of the carbons was observed. For example, in the case of CV adsorption on CQW-CO_2_ and CQW-H_2_O carbons, very similar adsorption values (12.67 vs. 11.77 mg L dye/mg charcoal) were obtained. However, the surface area of the steam-activated sample (798 m^2^/g) quadruples that activated with CO_2_ (199 m^2^/g). This result would indicate that the adsorption in the studied samples is governed by the interactions of weak (van der Waals forces) and strong (electrostatic interactions) [70], and that the generated porosity did not have an essential role in the adsorption process. The adsorption kinetics studies later corroborate what has been indicated by obtaining a better correlation to the pseudo-second-order kinetics. This result suggests that the chemisorption mechanism dominates the sorption process [71]. In addition, the FT-IR results will show the presence of hydroxyl and carboxylic groups on the surface of the carbons that are considered responsible for CV adsorption [72].

According to the statistical analysis, the experimental conditions in which the optimum conditions are obtained for each type of charcoal used correspond to the longest contact time (120 min) and a higher concentration of dye (400 mg/L). The results of the dye capture experiments, model equations, Pareto charts, and estimated response surface can be found in the supplementary data section (See Tables S1–S27 and Figures S1–S9).

We only focused the characterization discussion on QS-CO_2_, QS-H_2_O, CQW-CO_2_, and CQW-H_2_O because these carbons showed the best adsorption efficiency, as discussed above.

### 3.3. Docking-Based Interactions Analysis

**Figure 5:**
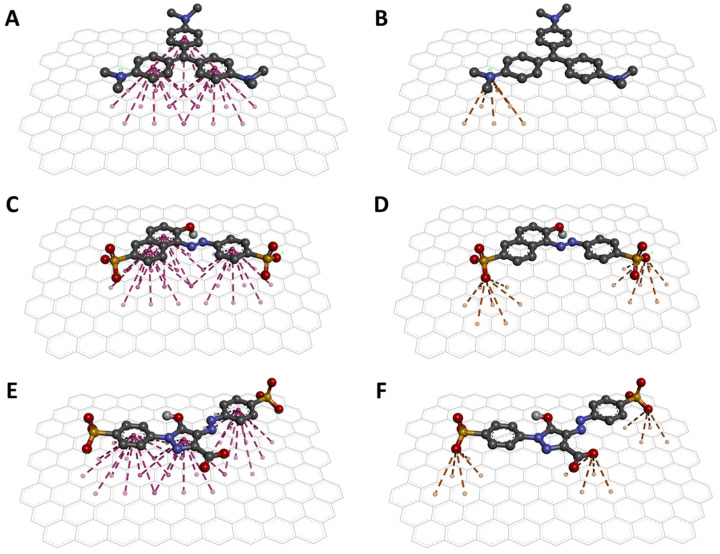
Interaction models of the dyes on the AC surface. π-stacking (**A**) and cation-π (**B**) interactions between CV and AC. π-stacking (**C**) and anion-π (**D**) interactions between SSY and AC. π-stacking (**E**) and anion-π (**F**) interactions between TAR and AC.

Molecular docking revealed the interaction between the dyes and the AC surface. The binding energies obtained from the best docking poses indicate that CV has a higher affinity for AC with a binding energy of −16.2 kcal/mol. As for the other two dyes, the binding energies obtained were −12.9 and −11.4 kcal/mol for SSY and TAR, respectively. These values suggest that CV would be the most absorbed dye by AC and that SSY and TAR should be absorbed similarly on AC surfaces. Then, the conformations of the best poses are shown in Figure 5. First, the torsions detected during docking allowed the dyes to adopt planar conformations to favor interactions. The three aromatic rings can form three sets of π-stacking interactions with the AC surface (Figure 5A,C,E).

Additionally, ion-π interactions were detected. For the case of CV, a cation-π interaction can be observed, formed between the charged amine and the surface of AC (Figure 5B). For SSY and TAR, interactions of the anion-π type (Figure 5D,F) were detected between the sulfate groups and the AC, and specific anion-π interaction between the carboxylic acid of TAR and AC (Figure 5F). So far, it has been reported that π-stacking interactions are fundamental in the absorption of dyes in AC and other carbon materials [73,74,75]. However, the present study shows an important contribution of the cation-π interaction to achieve a better affinity of the cationic dye over the anionic dyes. This observation is supported by the energy contribution of each of these interactions. First, individual cation-π interactions can contribute 2 to 5 kcal/mol to the binding of 2 molecules [76]. In contrast, experimental measurements of the energy of a single anion-π interaction suggest that such interactions contribute approximately 0.5 kcal/mol [77]. These results explain the excellent absorption of the AC against the CV dye in the experimental analyses.

### 3.4. Surface Analysis of QS and CQW Activated by CO_2_ and Steam before and after the CV Dye Adsorption

**Figure 6:**
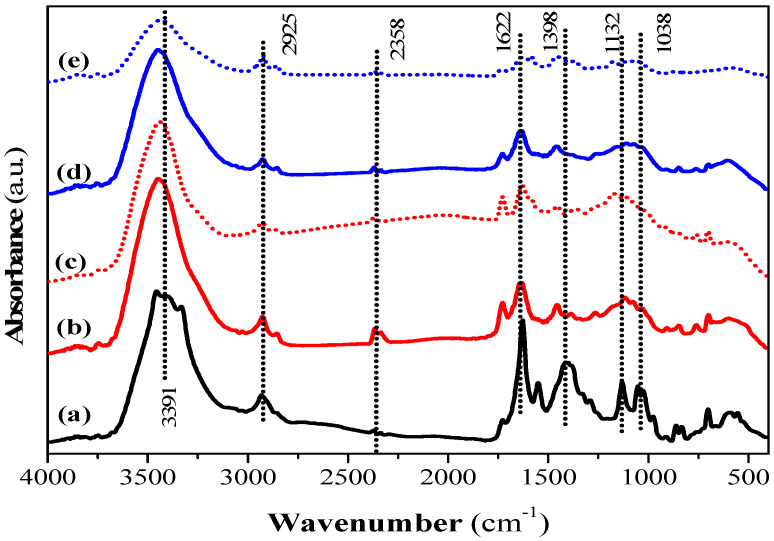
FT-IR spectra of QS-P (**a**), QS-CO_2_ (**b**), QS-CO_2_ CV (**c**), QS-H_2_O (**d**), and QS-H_2_O CV (**e**).

Surface functional groups on the QS and CQW activated by CO_2_ and steam were characterized by a Fourier transform infrared spectrometer and X-ray absorptiometry before and after dye adsorption. The FT-IR spectra of QS-P, QS-CO_2_, QS-CO_2_ CV, QS-H_2_O, and QS-H_2_O CV are shown in Figure 6. All FT-IR spectra showed the characteristic bands found in the carbonaceous materials. The bands observed at 3391, 2925, and 2358 cm^−1^ can be assigned to O-H stretching of hydroxyl groups [78], to C-H stretching or deformation from CH_2_ to CH_3_ [79], and to C-H stretching due to the presence of CH_2_-CO-group [78], respectively. The band at 1622 cm^−1^ is related to C=O and C-O stretching of carboxylic acid [80], while the band at 1398 cm^−1^ corresponds to C-O stretching vibration from the carboxyl group [41]. The bands at 1132 and 1038 cm^−1^ were attributed to C-C, C-O, or C-H from the carboxyl groups (-COOH) [41] and to the C-O group in carboxylic and alcoholic groups [81], respectively. In addition, the peaks below 1000 cm^−1^ were related to aromatic, out-of-plane C-H bending with different degrees of substitution [79]. These results showed the presence of hydroxyl and carboxyl groups that have been reported responsible for CV dye adsorption [72]. The main difference between QS-P and its related products generated by CO_2_ and steam activation was that some bands described above decreased in intensity or disappeared. This conclusion can be corroborated in the QS-CO_2_ and QS-H_2_O FT-IR spectra, respectively, indicating that the activation process was a success. Similar bands to those registered in the samples prepared in this investigation have been reported in commercial AC [82]. After the dye adsorption, it was possible to observe the same behavior commented before (See Figure 6c,e). This behavior was observed in Figure S10 (See Supplementary Materials) for the FT-IR spectra of CQW-P, CQW-CO_2_, CQW-CO_2_ CV, CQW-H_2_O, and CQW-H_2_O CV.

Finally, the results demonstrate that this material presented an ion exchange capacity and adsorption properties derived from its molecular structure.

**Figure 7:**
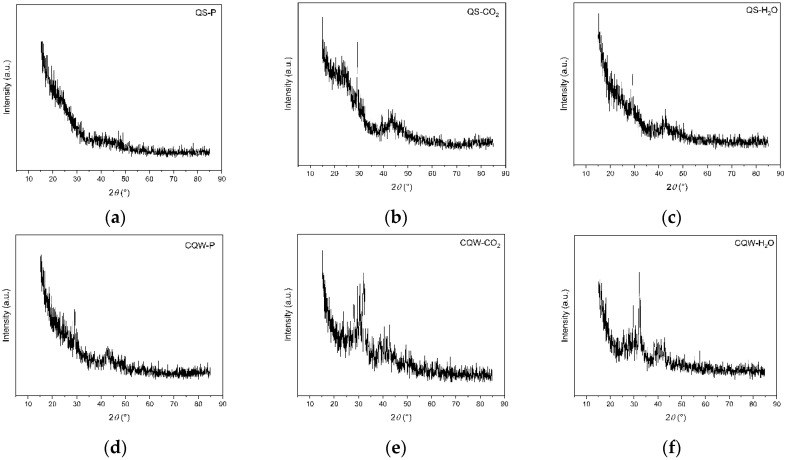
XRD patterns spectrum of QS-P (**a**), QS-CO_2_ (**b**), QS-H_2_O (**c**), CQW-P (**d**), CQW-CO_2_ (**e**), and CQW-H_2_O (**f**).

XRD patterns of QS-P, QS-CO_2_, QS-H_2_O, CQW-P, CQW-CO_2_, and CQW-H_2_O are shown in Figure 7. As shown in Figure 7, all samples exhibited an amorphous characteristic. Note that the activation process affects the amorphous percentage, where the carbonaceous materials from QS are the most affected. Specifically, the amorphous percentage values for QS-P, QS-CO_2_, and QS-H_2_O were 100, 52.2, and 49.9, respectively. In the cases of CQW-P, CQW-CO_2_, and CQW-H_2_O, the values were 50.3, 46.4, and 38.6, respectively. This behavior is because, during the activation process, the levels of the crystalline phase present in the pyrolyzed carbon changed significantly, forming fewer crystalline compounds. This behavior agreed with the results obtained by Grigore et al. [83].

**Figure 8:**
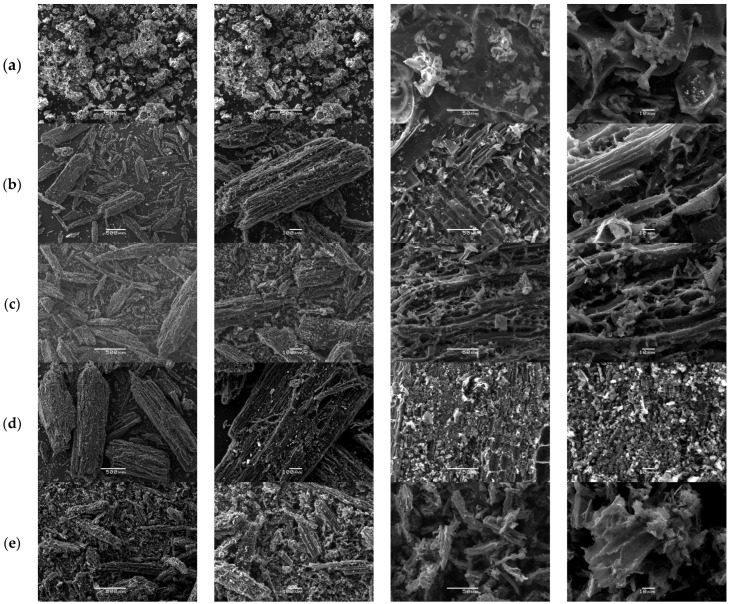
SEM images of the QS-P (**a**), QS-CO_2_ (**b**), QS-CO_2_ CV (**c**), QS-H_2_O (**d**), and QS-H_2_O CV (**e**). All images were taken with a magnification of 500×, 100×, 50×, and 10×, respectively.

Following the same protocol, the morphology of QS-P, QS-CO_2_, QS-H_2_ CV, QS-H_2_O, and QS-H_2_O CV surfaces at different SEM magnifications is illustrated in Figure 8. All figures show that the adsorbent obtained from QS had a particulate form with irregular shapes. Specifically, Figure 8a shows some macropores in the surface of QS-P derived from the natural structures of QS. Furthermore, after activation for both methods, we can appreciate the formation of a heterogeneous surface with abundant empty spaces and high porosity (see Figure 8b,e), similar to that observed in the commercial AC [47]. These results indicate that CO_2_ and steam can be effective activators for carbon materials derived from quinoa. The obtained results agree with the results reported by Molina-Sabio et al. [84].

Furthermore, it is known that forming these pores can be considered a desirable property that could increase the capacity of QS-CO_2_ and QS-H_2_O to absorb CV dyes. After the QS-CO_2_ and QS-H_2_O were allowed to adsorb the CV, the SEM images were taken and analyzed. Based on the analysis of the photos taken by SEM before and after the adsorption process, it was observed that the adsorption has the form of a thin and uniform layer (see Figure 8c,e). These results were recognizable by the disappearance of the distinguishable relief and decreased porosity of the activated carbon used.

The same behavior occurs with CQW-P, CQW-CO_2_, CQW-CO_2_ CV, CQW-H_2_O, and CQW-H_2_O CV. For that reason, we do not discuss the analysis of the images in the main text. Finally, the morphology of the CQW-P, CQW-CO_2_, CQW-CO_2_ CV, CQW-H_2_O, and CQW-H_2_O CV surfaces at different SEM magnifications is illustrated in Figure S11 (See Supplementary Materials).

### 3.5. Equilibrium Isotherm Modeling

**Figure 9:**
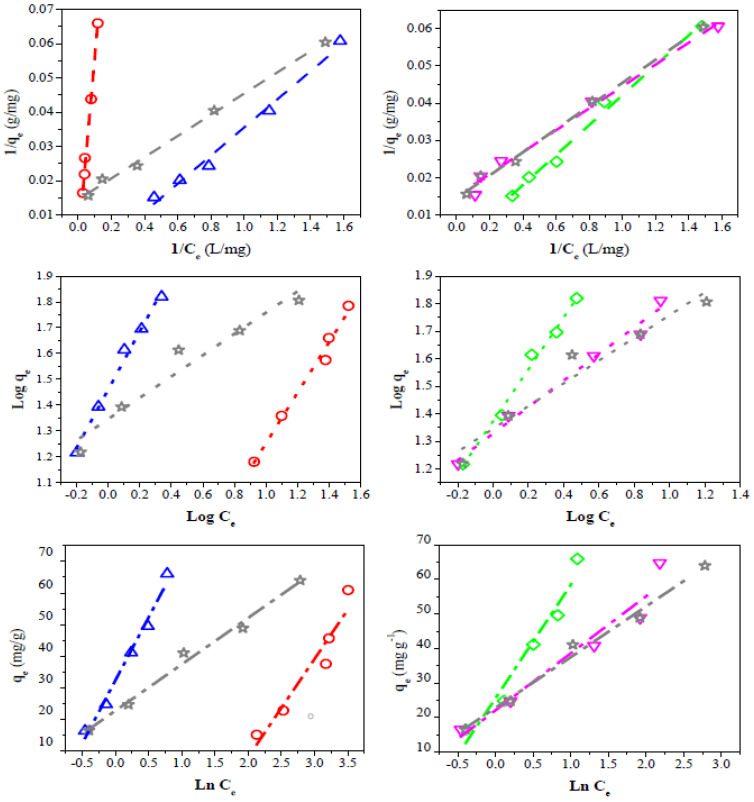
Linear fit of experimental data to different isotherm adsorption models. Langmuir (dash line), Freundlich (dot line), Temkin (dash-dot line). QS-H_2_O (**◇**), QS-CO_2_ (**▽**), CQW-CO_2_ (**☆**), CQW-P (**△**), and AC (**◯**).

To successfully represent the equilibrium adsorption behavior, it is important to have a satisfactory description of the equation state between the two phases composing the adsorption system. Therefore, three kinds of several isotherm equations were tested to fit the experimental data. The linearized Langmuir, Freundlich, and Temkin isotherms of dye are shown in Figure 9, while the estimated isotherm parameters are presented in Table 5.

The Langmuir model considers homogeneous adsorption, in which the adsorption activation energy of each sorbate molecule on the surface is equal [85]. The R^2^ and R_L_ values obtained for the Langmuir isotherm suggest that this model represents the CV dye adsorption. However, the sample QS-H_2_O registered a negative q_max_ value. This behavior can be related to the adsorption process’s electrostatic character [86]. The high q_max_ values observed for the QS-CO_2_ (854 mg/g) and CQW-CO_2_ (490 mg/g) samples are noticeable, which do not correlate with the data of capture dye capability reported in Table 4. However, given the possibility of dye adsorption on the carbon surface in mono- and multilayers, these values may be overestimated. This suggestion will be discussed later.

The Freundlich equation represents heterogeneous systems and reversible adsorption, and it is not limited to monolayer formation [87]. For the Freundlich adsorption model, the R^2^ parameter was similar to that reported for the Langmuir model. The *n* values obtained (*n* > 1) indicate that the adsorption conditions are favorable [88]. The experimental data fit better with these last two models than with the Temkin equation. The Temkin isotherm considers the existence of adsorbate-adsorbate interactions in the adsorption process. It is applicable in a wide range of ion concentrations [89]. The R^2^ parameter of the evaluated model follows the order: Temkins < Langmuir ~ Freundlich. This result indicates that these two models can describe the adsorption equilibrium. Therefore, the CV adsorption would occur on the carbon surface in both monolayers and multilayers. Similar results were reported for the CV adsorption using coffee husks and the reactive dyes on activated sludge [88,90].

### 3.6. Kinetics Analysis

**Figure 10:**
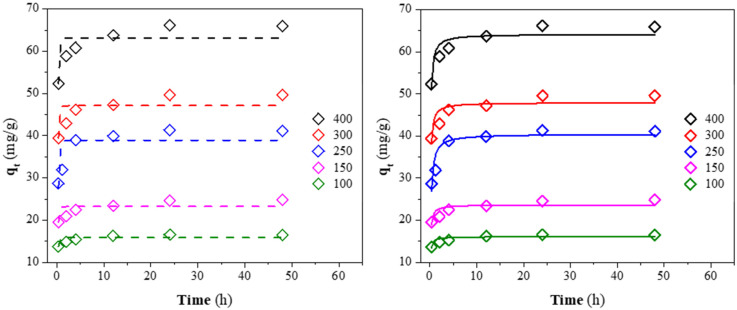
Pseudo-first (dash line) and second-order (solid line) kinetic fit for CV on CQW-CO_2_ at different initial concentrations.

The non-linear fit of the kinetic data for the CV adsorption at different initial concentrations is presented in Figure 10. The kinetics parameters for the pseudo-first and second-order models are reported in Table 6. The high correlation coefficient (R^2^) and low Chi-square coefficient (*χ*^2^) obtained for the pseudo-second-order rate indicate that this model explains the adsorption kinetics better. In addition, the good agreement between the calculated (*q_e,c_*) and experimental (*q_e_*) results of the dye capacity adsorption demonstrates that CV adsorption onto CQW-CO_2_ followed the kinetic mentioned above. The fit to this kinetic indicates that the chemisorption is the controlling step in the CV adsorption onto CQW-CO_2_ [88]. Similar results have been reported in other investigations [40,91]. From the data in Table 6, a decrease in the constant rate of *k*_2_ can be observed as the initial dye concentration increases. It is explained by the intense competition for sorption surface sites at high concentrations, which increases sorption rates.

## 4. Conclusions

This study investigated the use of a low-cost material used to produce an activated carbon derived from quillay and quinoa, for the elimination of two organic anionics (tartrazine (TAR) and sunset yellow FCF (SSY)), and cationic dye (crystal violet (CV)), from simulated dye polluted water. The samples were activated using CO_2_ and steam. The results obtained from the proximal and final analysis of the activated carbons show that both activation processes were successful. Moreover, the steam-activated samples exhibited a high total pore volume with a BET (Brunauer–Emmett–Teller theory) surface area of around 800 m^2^ g^−1^. On the other hand, the best NH_3_ adsorption capacity was reported for QS-H_2_O. Batch adsorption experiments showed that AC was the charcoal that offered the best adsorption efficiency for TAR and SSY, to the detriment of those obtained in this study. However, in the case of CV, all activated carbons obtained from CQW and QS offered the best captures, outperforming AC. Specifically, QS-CO_2_, QS-H_2_O, CQW-CO_2_, and CQW-H_2_O showed adsorption values of 8.99, 11.21, 11.77, and 12.67 mg/L CV/mg charcoal, respectively. This behavior was in agreement with the theoretical results using molecular dockings (AutoDock Vina). These activated carbons were fully characterized by SEM (scanning electron microscopy), FT-IR (Fourier transform infrared), and XRD (X-ray diffraction). The FT-IR spectrum of all samples showed the characteristic functional groups found in the carbonaceous materials. Moreover, it confirmed that the activation and dye adsorption were successful due to the difference in the FT-IR spectra patterns (some bands described above decreased in intensity or disappeared). The XRD patterns of all samples presented an amorphous characteristic. It is essential to mention that the activation process affects the amorphous percentage, as the carbonaceous QS materials are the most affected. Moreover, the SEM morphology of all samples exhibited a porous structure favorable for dye capture. Finally, the Langmuir, Freundlich, and Temkin isotherm models fit the adsorption data well. The kinetic results of the three isotherms models for the present data follow the order: Langmuir~Freundlich > Temkin. The promising results concluded that activated carbon obtained from quillay and quinoa could be potentially utilized to remove CV dyes. The promising results concluded that activated carbon obtained from quillay and quinoa could be potentially utilized to remove CV, TAR, and SSY dyes.

## Figures and Tables

**Table 1:** Experimental design of dye adsorption experiments.

Time-min	Dye Concentration-mg/L
120 (1)	400 (1)
20 (−1)	400 (1)
120 (1)	100 (−1)
20 (−1)	100 (−1)
70 (0)	250 (0)
70 (0)	250 (0)
70 (0)	250 (0)

**Table 2:** Proximate and ultimate analysis of raw, pyrolyzed, and activated carbons.

Sample	*Proximate Analysis-wt. %*				*Ultimate Analysis-wt. %*		
	Moisture	Ash	Volatiles	Fixed Carbon ^a^	C	H	N
CQW	10.84	15.19	84.49	0.32	47.74	6.33	1.63
QS	10.36	2.26	87.93	9.81	49.20	6.32	0.40
CQW-P	8.54	44.05	33.98	21.97	50.57	2.33	1.68
QS-P	3.45	3.46	28.38	68.15	84.55	3.32	0.95
CQW-CO_2_	10.11	50.03	28.92	21.05	46.08	0.96	1.89
QS-CO_2_	5.39	10.74	14.69	74.57	86.69	1.22	1.34
CQW-H_2_O	0.79	71.39	19.48	9.13	31.93	0.96	0.65
QS-H_2_O	0.49	12.29	18.12	69.59	83.04	0.90	0.98

^a^ Dry basis.

**Table 3:** Textural properties, yield, pH, and NH_3_ adsorption capacity of pyrolytic and physical activated samples.

Sample	S_BET_-m^2^/g	V_tot_-cm^3^/g	D ^a^-nm	Y-%	pH	Iodine Number-mg/g	NH_3_ Adsorption Capacity-mmol/g
QS-P	3	0.006	7.92	26.0	7.98	119	4.14
QS-CO_2_	479	0.219	1.83	21.1	8.14	294	6.50
QS-H_2_O	813	0.420	2.06	17.2	10.61	619	6.50
CQW-P	9	0.039	14.08	28.2	11.34	215	2.74
CQW-CO_2_	199	0.135	2.52	19.0	11.07	279	0.91
CQW-H_2_O	798	0.395	1.90	20.2	11.42	522	5.03

S_BET_: BET surface area; V_tot_: total pore volume; D: average pore diameter. ^a^ 4 × V_tot_/S_BET_.

**Table 4:** Comparison between the capture capabilities of the different types of charcoal for dye solutions.

Dye	Sample	Maximum Dye Remotion- mg/L dye/mg Charcoal
Tartrazine	AC	9.65
	QS-P	6.68
	QS-CO_2_	3.22
	QS-H_2_O	8.01
	CQW-P	8.08
	CQW-CO_2_	7.55
	CQW-H_2_O	7.26
Crystal violet	AC	7.06
	QS-P	3.11
	QS-CO_2_	8.99
	QS-H_2_O	11.21
	CQW-P	6.60
	CQW-CO_2_	11.77
	CQW-H_2_O	12.67
Sunset yellow FCF	AC	10.44
	QS-P	4.21
	QS-CO_2_	4.45
	QS-H_2_O	8.13
	CQW-P	7.21
	CQW-CO_2_	6.66
	CQW-H_2_O	6.96

**Table 5:** Model parameters calculated for CV adsorption onto QS and CQW activated carbons.

Sample	*Langmuir*				*Freundlich*			*Temkin*		
	q_max_- mg/g	K_L_- L/mg	R_L_	R^2^	K_F_- (mg g^−1^)*(L mg^−1^)^n^	*n*	R^2^	a	b	R^2^
QS-H_2_O	−181.16	−0.1345	−0.0189	0.986	28.7614	0.89	0.987	32.342	39.856	0.981
QS-CO_2_	854.70	0.0021	0.5374	0.992	1.8745	1.02	0.983	−53.985	31.057	0.903
CQW-CO_2_	490.19	0.0509	0.0468	0.989	23.6037	1.06	0.988	25.543	32.817	0.942
CQW-P	65.96	0.5172	0.0048	0.985	21.2956	2.08	0.981	22.059	16.517	0.926
AC	69.15	0.4667	0.0053	0.995	22.1554	2.42	0.931	22.772	14.691	0.985

**Table 6:** Kinetic parameters for CV adsorption on QS, CQW, and AC samples.

Sample	*C*_0_- mg/L	*q_e_*- mg/g	*Pseudo-First Order Model*				*Pseudo-Second Order Model*			
			*q_e,c_*- mg/g	*k*_1_- h^−1^	R^2^	*χ* ^2^	*q_e,c_*- mg/g	*k*_2_- mg/(g h)	R^2^	*χ* ^2^
QS-H_2_O	100	16.45	16.46	10.917	0.7678	0.0089	16.49	6.1559	0.8500	0.0057
	150	24.77	24.67	6.341	0.9883	0.0174	24.88	0.8327	0.9923	0.0114
	250	41.18	38.91	3.758	0.4680	16.0266	40.55	0.1457	0.8130	5.6329
	300	49.56	47.07	4.479	0.5495	13.0872	48.31	0.1689	0.7646	6.8389
	400	66.08	62.72	4.468	0.5936	20.0224	64.33	0.1281	0.8009	9.8069
QS-CO_2_	100	15.16	14.22	6.4266	0.2156	0.9785	14.42	1.1921	0.4079	0.7386
	150	22.83	19.99	3.6234	0.3775	7.2766	20.88	0.2369	0.6122	4.5328
	250	37.57	32.95	4.6316	0.1851	17.7727	34.16	0.2302	0.4486	12.0236
	300	45.68	40.77	3.6749	0.4330	24.4524	42.51	0.1205	0.6849	13.5903
	400	60.91	53.50	3.9003	0.3003	55.7032	55.73	0.0984	0.5394	36.6687
CQW-CO_2_	100	16.50	15.89	5.9857	0.5160	0.6141	16.11	0.9654	0.7169	0.3592
	150	24.81	23.23	5.4720	0.4139	2.5839	23.67	0.5141	0.6311	1.6262
	250	41.12	38.89	3.7271	0.5177	13.8297	40.47	0.1485	0.8497	4.3098
	300	49.62	47.11	5.4328	0.5194	7.7132	47.93	0.2587	0.7223	4.4561
	400	65.95	63.09	5.2964	0.6299	10.2077	64.18	0.1869	0.8100	5.2392
CQW-P	100	16.51	16.39	8.6822	0.8979	0.0152	16.46	2.8637	0.9676	0.0048
	150	24.71	24.23	7.6611	0.7543	0.1810	24.38	1.2923	0.8844	0.0852
	250	40.78	40.78	7.6414	0.9821	0.0308	41.04	0.8376	0.9441	0.0963
	300	48.86	48.27	8.3843	0.8689	0.2124	48.49	0.8659	0.9571	0.0695
	400	64.75	64.33	7.4949	0.9169	0.4149	64.72	0.4754	0.9871	0.0644
AC	100	16.55	16.06	2.4353	0.9154	0.7329	16.79	0.2100	0.9895	0.0910
	150	24.71	23.37	0.5264	0.7675	9.0488	24.49	0.0405	0.9089	3.5443
	250	41.06	38.36	0.7627	0.7223	31.6107	40.80	0.0283	0.8867	12.9003
	300	48.86	46.69	0.4442	0.9161	16.1052	49.79	0.0144	0.9686	6.0317
	400	63.97	61.87	0.4512	0.9247	25.6784	65.96	0.0110	0.9709	9.8885

## Data Availability

Not applicable.

## Supplementary Materials

The following supporting information can be downloaded at: https://www.mdpi.com/article/10.3390/ma15144898/s1, Table S1. QS charcoal used as tartrazine removal treatment (numbers in parentheses correspond to the coded values used in the statistical analysis); Table S2. CQW charcoal used as tartrazine removal treatment (numbers in parentheses correspond to the coded values used in the statistical analysis); Table S3. QS-CO_2_ used as tartrazine removal treatment (numbers in parentheses correspond to the coded values used in the statistical analysis); Table S4. CQW-CO_2_ used as tartrazine removal treatment (numbers in parentheses correspond to the coded values used in the statistical analysis); Table S5. QS-H_2_O used as tartrazine removal treatment (numbers in parentheses correspond to the coded values used in the statistical analysis); Table S6. CQW-H_2_O used as tartrazine removal treatment (numbers in parentheses correspond to the coded values used in the statistical analysis); Table S7. QS-P used as tartrazine removal treatment (numbers in parentheses correspond to the coded values used in the statistical analysis); Table S8. CQW-P used as tartrazine removal treatment (numbers in parentheses correspond to the coded values used in the statistical analysis); Table S9. AC used as tartrazine removal treatment (numbers in parentheses correspond to the coded values used in the statistical analysis); Table S10. QS charcoal used as crystal violet removal treatment (numbers in parentheses correspond to the coded values used in the statistical analysis); Table S11. CQW charcoal used as crystal violet removal treatment (numbers in parentheses correspond to the coded values used in the statistical analysis); Table S12. QS-CO_2_ used as crystal violet removal treatment (numbers in parentheses correspond to the coded values used in the statistical analysis); Table S13. CQW-CO_2_ used as crystal violet removal treatment (numbers in parentheses correspond to the coded values used in the statistical analysis); Table S14. QS-H_2_O used as crystal violet removal treatment (numbers in parentheses correspond to the coded values used in the statistical analysis); Table S15. CQW-H_2_O used as crystal violet removal treatment (numbers in parentheses correspond to the coded values used in the statistical analysis); Table S16. QS-P used as crystal violet removal treatment (numbers in parentheses correspond to the coded values used in the statistical analysis); Table S17. CQW-P used as crystal violet removal treatment (numbers in parentheses correspond to the coded values used in the statistical analysis); Table S18. AC used as crystal violet removal treatment (numbers in parentheses correspond to the coded values used in the statistical analysis); Table S19. QS charcoal used as sunset yellow FCF removal treatment (numbers in parentheses correspond to the coded values used in the statistical analysis); Table S20. CQW charcoal used as sunset yellow FCF removal treatment (numbers in parentheses correspond to the coded values used in the statistical analysis); Table S21. QS-CO_2_ used as sunset yellow FCF removal treatment (numbers in parentheses correspond to the coded values used in the statistical analysis); Table S22. CQW-CO_2_ used as sunset yellow FCF removal treatment (numbers in parentheses correspond to the coded values used in the statistical analysis); Table S23. QS-H_2_O used as sunset yellow FCF removal treatment (numbers in parentheses correspond to the coded values used in the statistical analysis); Table S24. CQW-H_2_O used as sunset yellow FCF removal treatment (numbers in parentheses correspond to the coded values used in the statistical analysis); Table S25. QS-P used as sunset yellow FCF removal treatment (numbers in parentheses correspond to the coded values used in the statistical analysis); Table S26. CQW-P used for sunset yellow FCF removal treatment (numbers in parentheses correspond to the coded values used in the statistical analysis); Table S27. AC used as sunset yellow FCF removal treatment (numbers in parentheses correspond to the coded values used in the statistical analysis); Figure S1. (a), Standardized Pareto chart for tartrazine remotion by AC (where: A, time of interaction; B, tartrazine concentration; AB, interaction. The line represents the critical *t*-value, 95% confidence); (b), Estimated response surface; Figure S2. (a–d): Standardized Pareto charts for tartrazine remotion by QS, QS-CO_2_, QS-H_2_O, and QS-P, respectively (where: A, time of interaction; B, tartrazine concentration; AB, interaction. The line represents the critical *t*-value, 95% confidence); (e–h): Estimated response surfaces for tartrazine capture by QS, QS-CO_2_, QS-H_2_O, and QS-P, respectively; Figure S3. (a–d): Standardized Pareto charts for tartrazine remotion by CQW, CQW-CO_2_, CQW-H_2_O, and CQW-P, respectively (where: A, time of interaction; B, tartrazine concentration; AB, interaction. The line represents the critical *t*-value, 95% confidence); (e–h): Estimated response surfaces for tartrazine capture by CQW, CQW-CO_2_, CQW-H_2_O, and CQW-P, respectively; Figure S4. (a), Standardized Pareto chart for crystal violet remotion by AC (where: A, time of interaction; B, crystal violet concentration; AB, interaction. The line represents the critical *t*-value, 95% confidence); (b), Estimated response surface; Figure S5. (a–d): Standardized Pareto charts for crystal violet remotion by QS, QS-CO_2_, QS-H_2_O, and QS-P, respectively (where: A, time of interaction; B, crystal violet concentration; AB, interaction. The line represents the critical *t*-value, 95% confidence); (e–h): Estimated response surfaces for crystal violet capture by QS, QS-CO_2_, QS-H_2_O, and QS-P, respectively; Figure S6. (a–d): Standardized Pareto charts for crystal violet remotion by CQW, CQW-CO_2_, CQW-H_2_O, and CQW-P, respectively (where: A, time of interaction; B, crystal violet concentration; AB, interaction. The line represents the critical *t*-value, 95% confidence); (e–h): Estimated response surfaces for crystal violet capture by CQW, CQW-CO_2_, CQW-H_2_O, and CQW-P, respectively; Figure S7. (a), Standardized Pareto chart for sunset yellow FCF remotion by AC (where: A, time of interaction; B, sunset yellow FCF concentration; AB, interaction. The line represents the critical *t*-value, 95% confidence); (b), Estimated response surface; Figure S8. (a–d): Standardized Pareto charts for sunset yellow FCF remotion by QS, QS-CO_2_, QS-H_2_O, and QS-P, respectively (where: A, time of interaction; B, sunset yellow FCF concentration; AB, interaction. The line represents the critical *t*-value, 95% confidence); (e–h): Estimated response surfaces for sunset yellow FCF capture by QS, QS-CO_2_, QS-H_2_O, and QS-P, respectively; Figure S9. (a–d): Standardized Pareto charts for sunset yellow FCF remotion by CQW, CQW-CO_2_, CQW-H_2_O, and CQW-P, respectively (where: A, time of interaction; B, sunset yellow FCF concentration; AB, interaction. The line represents the critical *t*-value, 95% confidence); (e–h): Estimated response surfaces for sunset yellow FCF capture by CQW, CQW-CO_2_, CQW-H_2_O, and CQW-P, respectively; Figure S10. FT-IR spectra of CQW-P (a), CQW-CO_2_ (b), CQW-CO_2_ CV (c), CQW-H_2_O (d), and CQW-H_2_O CV (e); Figure S11. SEM images of the CQW-P (a), CQW-CO_2_ (b), CQW-CO_2_ CV (c), CQW-H_2_O (d), and CQW-H_2_O CV (e). All images were taken with a magnification of 500×, 100×, 50×, and 10×, respectively.
