# Supplementary material for: Comparative Study of Three Dyes’ Adsorption onto Activated Carbon from Chenopodium quinoa Willd and Quillaja saponaria
Source: Materials (Basel). 2022 Jul 14;15(14):4898. doi: 10.3390/ma15144898 (PMC9321238; doi:10.3390/ma15144898)
Supplement: Supplementary file 1 [file materials-15-04898-s001.zip › materials-1784744-supplementary.pdf]

materials

Supplementary Materials: Table S1. QS charcoal used as tartrazine removal treatment (numbers in parentheses correspond to the coded values used in the statistical analysis); Table S2. CQW charcoal used as tartrazine removal treatment (numbers in parentheses correspond to the coded values used in the statistical analysis); Table S3. QS-CO_2 used as tartrazine removal treatment (numbers in parentheses correspond to the coded values used in the statistical analysis); Table S4. CQW-CO_2 used as tartrazine removal treatment (numbers in parentheses correspond to the coded values used in the statistical analysis); Table S5. QS-H_2O used as tartrazine removal treatment (numbers in parentheses correspond to the coded values used in the statistical analysis); Table S6. CQW-H_2O used as tartrazine removal treatment (numbers in parentheses correspond to the coded values used in the statistical analysis); Table S7. QS-P used as tartrazine removal treatment (numbers in parentheses correspond to the coded values used in the statistical analysis); Table S8. CQW-P used as tartrazine removal treatment (numbers in parentheses correspond to the coded values used in the statistical analysis); Table S9. AC used as tartrazine removal treatment (numbers in parentheses correspond to the coded values used in the statistical analysis); Table S10. QS charcoal used as crystal violet removal treatment (numbers in parentheses correspond to the coded values used in the statistical analysis); Table S11. CQW charcoal used as crystal violet removal treatment (numbers in parentheses correspond to the coded values used in the statistical analysis); Table S12. QS-CO_2 used as crystal violet removal treatment (numbers in parentheses correspond to the coded values used in the statistical analysis); Table S13. CQW-CO_2 used as crystal violet removal treatment (numbers in parentheses correspond to the coded values used in the statistical analysis); Table S14. QS-H_2O used as crystal violet removal treatment (numbers in parentheses correspond to the coded values used in the statistical analysis); Table S15. CQW-H_2O used as crystal violet removal treatment (numbers in parentheses correspond to the coded values used in the statistical analysis); Table S16. QS-P used as crystal violet removal treatment (numbers in parentheses correspond to the coded values used in the statistical analysis); Table S17. CQW-P used as crystal violet removal treatment (numbers in parentheses correspond to the coded values used in the statistical analysis); Table S18. AC used as crystal violet removal treatment (numbers in parentheses correspond to the coded values used in the statistical analysis); Table S19. QS charcoal used as sunset yellow FCF removal treatment (numbers in parentheses correspond to the coded values used in the statistical analysis); Table S20. CQW charcoal used as sunset yellow FCF removal treatment (numbers in parentheses correspond to the coded values used in the statistical analysis); Table S21. QS-CO_2 used as sunset yellow FCF removal treatment (numbers in parentheses correspond to the coded values used in the statistical analysis); Table S22. CQW-CO_2 used as sunset yellow FCF removal treatment (numbers in parentheses correspond to the coded values used in the statistical analysis); Table S23. QS-H_2O used as sunset yellow FCF removal treatment (numbers in parentheses correspond to the coded values used in the statistical analysis); Table S24. CQW-H_2O used as sunset yellow FCF removal treatment (numbers in parentheses correspond to the coded values used in the statistical analysis); Table S25. QS-P used as sunset yellow FCF removal treatment (numbers in parentheses correspond to the coded values used in the statistical analysis); Table S26. CQW-P used for sunset yellow FCF removal treatment (numbers in parentheses correspond to the coded values used in the statistical analysis); Table S27. AC used as sunset yellow FCF removal treatment (numbers in parentheses correspond to the coded values used in the statistical analysis); Figure S1. (a), Standardized Pareto chart for tartrazine remotion by AC (where: A, time of interaction; B, tartrazine concentration; AB, interaction. The line represents the critical *t*-value, 95% confidence); (b), Estimated response surface; Figure S2. (a–d): Standardized Pareto charts for tartrazine remotion by QS, QS-CO_2, QS-H_2O, and QS-P, respectively (where: A, time of interaction; B, tartrazine concentration; AB, interaction. The line represents the critical *t*-value, 95% confidence); (e–h): Estimated response surfaces for tartrazine capture by QS, QS-CO_2, QS-H_2O, and QS-P, respectively; Figure S3. (a–d): Standardized Pareto charts for tartrazine remotion by CQW, CQW-CO_2, CQW-H_2O, and CQW-P, respectively (where: A, time of interaction; B, tartrazine concentration; AB, interaction. The line represents the critical *t*-value, 95% confidence); (e–h): Estimated response surfaces for tartrazine capture by CQW, CQW-CO_2, CQW-H_2O, and CQW-P, respectively; Figure S4. (a), Standardized Pareto chart for crystal violet remotion by AC (where: A, time of interaction; B, crystal violet concentration; AB, interaction. The line represents the critical *t*-value, 95% confidence); (b), Estimated response surface; Figure S5. (a–d): Standardized Pareto charts for crystal violet remotion by QS, QS-CO_2, QS-H_2O, and QS-P, respectively (where: A, time of interaction; B, crystal violet concentration; AB, interaction. The line represents the critical *t*-value, 95% confidence); (e–h): Estimated response surfaces for crystal violet capture by QS, QS-CO_2, QS-H_2O, and QS-P, respectively; Figure S6. (a–d): Standardized Pareto charts for crystal violet remotion by CQW, CQW-CO_2, CQW-H_2O, and CQW-P, respectively (where: A, time of interaction; B,

crystal violet concentration; AB, interaction. The line represents the critical *t*-value, 95% confidence); (e–h): Estimated response surfaces for crystal violet capture by CQW, CQW-CO_2, CQW-H_2O, and CQW-P, respectively; Figure S7. (a), Standardized Pareto chart for sunset yellow FCF remotion by AC (where: A, time of interaction; B, sunset yellow FCF concentration; AB, interaction. The line represents the critical *t*-value, 95% confidence); (b), Estimated response surface; Figure S8. (a–d): Standardized Pareto charts for sunset yellow FCF remotion by QS, QS-CO_2, QS-H_2O, and QS-P, respectively (where: A, time of interaction; B, sunset yellow FCF concentration; AB, interaction. The line represents the critical *t*-value, 95% confidence); (e–h): Estimated response surfaces for sunset yellow FCF capture by QS, QS-CO_2, QS-H_2O, and QS-P, respectively; Figure S9. (a–d): Standardized Pareto charts for sunset yellow FCF remotion by CQW, CQW-CO_2, CQW-H_2O, and CQW-P, respectively (where: A, time of interaction; B, sunset yellow FCF concentration; AB, interaction. The line represents the critical *t*-value, 95% confidence); (e–h): Estimated response surfaces for sunset yellow FCF capture by CQW, CQW-CO_2, CQW-H_2O, and CQW-P, respectively; Figure S10. FT-IR spectra of CQW-P (a), CQW-CO_2 (b), CQW-CO_2 CV (c), CQW-H_2O (d), and CQW-H_2O CV (e); Figure S11. SEM images of the CQW-P (a), CQW-CO_2 (b), CQW-CO_2 CV (c), CQW-H_2O (d), and CQW-H_2O CV (e). All images were taken with a magnification of 500×, 100×, 50×, and 10×, respectively.

Tables:

Table S1. QS charcoal used as tartrazine removal treatment (numbers in parentheses correspond to the coded values used in the statistical analysis).

Time [min]	Tartrazine concentration [mg/L]	Charcoal mass [g]	mg/L Tartrazine captured/mg Charcoal
120 (1)	400 (1)	0.0302	2.37
20 (-1)	400 (1)	0.0303	1.14
120 (1)	100 (-1)	0.0304	1.33
20 (-1)	100 (-1)	0.0301	1.33
70 (0)	250 (0)	0.0304	2.01
70 (0)	250 (0)	0.0301	1.98
70 (0)	250 (0)	0.0300	2.00

Table S2. CQW charcoal used as tartrazine removal treatment (numbers in parentheses correspond to the coded values used in the statistical analysis).

Time [min]	Tartrazine concentration [mg/L]	Charcoal mass [g]	mg/L Tartrazine captured/mg Charcoal
120 (1)	400 (1)	0.0304	1.43
20 (-1)	400 (1)	0.0304	1.11
120 (1)	100 (-1)	0.0301	0.30
20 (-1)	100 (-1)	0.0300	0.06
70 (0)	250 (0)	0.0304	1.77
70 (0)	250 (0)	0.0300	1.84
70 (0)	250 (0)	0.0304	1.77

Table S3. QS-CO_2 used as tartrazine removal treatment (numbers in parentheses correspond to the coded values used in the statistical analysis).

Time [min]	Tartrazine concentration [mg/L]	Charcoal mass [g]	mg/L Tartrazine captured/mg Charcoal
120 (1)	400 (1)	0.0302	3.22
20 (-1)	400 (1)	0.0303	1.94
120 (1)	100 (-1)	0.0301	0.63
20 (-1)	100 (-1)	0.0303	0.62
70 (0)	250 (0)	0.0300	0.78
70 (0)	250 (0)	0.0300	0.85
70 (0)	250 (0)	0.0300	0.81

Table S4. CQW-CO_2 used as tartrazine removal treatment (numbers in parentheses correspond to the coded values used in the statistical analysis).

Time [min]	Tartrazine concentration [mg/L]	Charcoal mass [g]	mg/L Tartrazine captured/mg Charcoal
120 (1)	400 (1)	0.0300	7.55
20 (-1)	400 (1)	0.0301	7.30
120 (1)	100 (-1)	0.0301	1.79
20 (-1)	100 (-1)	0.0302	1.29
70 (0)	250 (0)	0.0304	3.36
70 (0)	250 (0)	0.0304	3.42
70 (0)	250 (0)	0.0304	3.36

Table S5. QS-H_2O used as tartrazine removal treatment (numbers in parentheses correspond to the coded values used in the statistical analysis).

Time [min]	Tartrazine concentration [mg/L]	Charcoal mass [g]	mg/L Tartrazine captured/mg Charcoal
120 (1)	400 (1)	0.0303	8.01
20 (-1)	400 (1)	0.0300	6.66
120 (1)	100 (-1)	0.0301	3.30
20 (-1)	100 (-1)	0.0304	2.85
70 (0)	250 (0)	0.0303	5.67
70 (0)	250 (0)	0.0301	5.69
70 (0)	250 (0)	0.0301	5.64

Table S6. CQW-H_2O used as tartrazine removal treatment (numbers in parentheses correspond to the coded values used in the statistical analysis).

Time [min]	Tartrazine concentration [mg/L]	Charcoal mass [g]	mg/L Tartrazine captured/mg Charcoal
120 (1)	400 (1)	0.0303	7.26
20 (-1)	400 (1)	0.0300	7.19
120 (1)	100 (-1)	0.0302	1.35
20 (-1)	100 (-1)	0.0302	1.01
70 (0)	250 (0)	0.0304	2.83
70 (0)	250 (0)	0.0300	2.99
70 (0)	250 (0)	0.0304	2.89

Table S7. QS-P used as tartrazine removal treatment (numbers in parentheses correspond to the coded values used in the statistical analysis).

Time [min]	Tartrazine concentration [mg/L]	Charcoal mass [g]	mg/L Tartrazine captured/mg Charcoal
120 (1)	400 (1)	0.0300	6.68
20 (-1)	400 (1)	0.0300	4.14
120 (1)	100 (-1)	0.0301	0.78
20 (-1)	100 (-1)	0.0303	0.57
70 (0)	250 (0)	0.0303	0.62
70 (0)	250 (0)	0.0301	0.60
70 (0)	250 (0)	0.0303	0.61

Table S8. CQW-P used as tartrazine removal treatment (numbers in parentheses correspond to the coded values used in the statistical analysis).

Time [min]	Tartrazine concentration [mg/L]	Charcoal mass [g]	mg/L Tartrazine captured/mg Charcoal
120 (1)	400 (1)	0.0300	8.08
20 (-1)	400 (1)	0.0301	7.16
120 (1)	100 (-1)	0.0302	1.04
20 (-1)	100 (-1)	0.0304	0.96
70 (0)	250 (0)	0.0304	2.95
70 (0)	250 (0)	0.0303	2.84
70 (0)	250 (0)	0.0304	2.86

Table S9. AC used as tartrazine removal treatment (numbers in parentheses correspond to the coded values used in the statistical analysis).

Time [min]	Tartrazine concentration [mg/L]	Charcoal mass [g]	mg/L Tartrazine captured/mg Charcoal
120 (1)	400 (1)	0.0300	9.65
20 (-1)	400 (1)	0.0303	6.41
120 (1)	100 (-1)	0.0300	3.05
20 (-1)	100 (-1)	0.0303	2.01
70 (0)	250 (0)	0.0301	6.25
70 (0)	250 (0)	0.0300	6.22
70 (0)	250 (0)	0.0301	6.33
120 (1)	400 (1)	0.0300	9.58
20 (-1)	400 (1)	0.0300	6.47
120 (1)	100 (-1)	0.0300	2.98
20 (-1)	100 (-1)	0.0304	2.02
70 (0)	250 (0)	0.0300	6.42
70 (0)	250 (0)	0.0304	6.39
70 (0)	250 (0)	0.0303	6.50

Table S10. QS charcoal used as crystal violet removal treatment (numbers in parentheses correspond to the coded values used in the statistical analysis).

Time [min]	Crystal Violet concentration [mg/L]	Charcoal mass [g]	mg/L Crystal Violet captured/mg Charcoal
120 (1)	400 (1)	0.0303	6.54
20 (-1)	400 (1)	0.0304	5.51
120 (1)	100 (-1)	0.0304	2.56
20 (-1)	100 (-1)	0.0300	2.45
70 (0)	250 (0)	0.0302	5.21
70 (0)	250 (0)	0.0301	5.21
70 (0)	250 (0)	0.0300	5.21

Table S11. CQW charcoal used as crystal violet removal treatment (numbers in parentheses correspond to the coded values used in the statistical analysis).

Time [min]	Crystal Violet concentration [mg/L]	Charcoal mass [g]	mg/L Crystal Violet captured/mg Charcoal
120 (1)	400 (1)	0.0302	4.16
20 (-1)	400 (1)	0.0300	3.19
120 (1)	100 (-1)	0.0303	1.36
20 (-1)	100 (-1)	0.0300	1.28
70 (0)	250 (0)	0.0302	4.71
70 (0)	250 (0)	0.0301	4.73
70 (0)	250 (0)	0.0302	4.71

Table S12. QS-CO_2 used as crystal violet removal treatment (numbers in parentheses correspond to the coded values used in the statistical analysis).

Time [min]	Crystal Violet concentration [mg/L]	Charcoal mass [g]	mg/L Crystal Violet captured/mg Charcoal
120 (1)	400 (1)	0.0300	8.99
20 (-1)	400 (1)	0.0303	7.80
120 (1)	100 (-1)	0.0300	2.62
20 (-1)	100 (-1)	0.0300	2.51
70 (0)	250 (0)	0.0300	5.72
70 (0)	250 (0)	0.0300	5.76
70 (0)	250 (0)	0.0300	5.72

Table S13. CQW-CO_2 used as crystal violet removal treatment (numbers in parentheses correspond to the coded values used in the statistical analysis).

Time [min]	Crystal Violet concentration [mg/L]	Charcoal mass [g]	mg/L Crystal Violet captured/mg Charcoal
120 (1)	400 (1)	0.0302	11.77
20 (-1)	400 (1)	0.0303	10.46
120 (1)	100 (-1)	0.0302	2.95
20 (-1)	100 (-1)	0.0302	2.74
70 (0)	250 (0)	0.0301	6.35
70 (0)	250 (0)	0.0300	6.44
70 (0)	250 (0)	0.0303	6.37

Table S14. QS-H_2O used as crystal violet removal treatment (numbers in parentheses correspond to the coded values used in the statistical analysis).

Time [min]	Crystal Violet concentration [mg/L]	Charcoal mass [g]	mg/L Crystal Violet captured/mg Charcoal
120 (1)	400 (1)	0.0301	11.21
20 (-1)	400 (1)	0.0304	9.72
120 (1)	100 (-1)	0.0301	3.26
20 (-1)	100 (-1)	0.0304	3.18
70 (0)	250 (0)	0.0300	7.41
70 (0)	250 (0)	0.0301	7.36
70 (0)	250 (0)	0.0300	7.39

Table S15. CQW-H_2O used as crystal violet removal treatment (numbers in parentheses correspond to the coded values used in the statistical analysis).

Time [min]	Crystal Violet concentration [mg/L]	Charcoal mass [g]	mg/L Crystal Violet captured/mg Charcoal
120 (1)	400 (1)	0.0300	6.60
20 (-1)	400 (1)	0.0301	4.37
120 (1)	100 (-1)	0.0302	2.24
20 (-1)	100 (-1)	0.0302	1.56
70 (0)	250 (0)	0.0304	3.48
70 (0)	250 (0)	0.0303	3.49
70 (0)	250 (0)	0.0300	3.58

Table S16. QS-P used as crystal violet removal treatment (numbers in parentheses correspond to the coded values used in the statistical analysis).

Time [min]	Crystal Violet concentration [mg/L]	Charcoal mass [g]	mg/L Crystal Violet captured/mg Charcoal
120 (1)	400 (1)	0.0302	3.11
20 (-1)	400 (1)	0.0300	1.03
120 (1)	100 (-1)	0.0301	0.77
20 (-1)	100 (-1)	0.0302	0.30
70 (0)	250 (0)	0.0300	2.26
70 (0)	250 (0)	0.0300	2.34
70 (0)	250 (0)	0.0304	2.29

Table S17. CQW-P used as crystal violet removal treatment (numbers in parentheses correspond to the coded values used in the statistical analysis).

Time [min]	Crystal Violet concentration [mg/L]	Charcoal mass [g]	mg/L Crystal Violet captured/mg Charcoal
120 (1)	400 (1)	0.0303	12.67
20 (-1)	400 (1)	0.0304	11.80
120 (1)	100 (-1)	0.0302	3.23
20 (-1)	100 (-1)	0.0300	3.09
70 (0)	250 (0)	0.0302	8.10
70 (0)	250 (0)	0.0301	8.11
70 (0)	250 (0)	0.0300	8.16

Table S18. AC used as crystal violet removal treatment (numbers in parentheses correspond to the coded values used in the statistical analysis).

Time [min]	Crystal Violet concentration [mg/L]	Charcoal mass [g]	mg/L Crystal Violet captured/mg Charcoal
120 (1)	400 (1)	0.0300	7.06
20 (-1)	400 (1)	0.0302	3.53
120 (1)	100 (-1)	0.0300	2.88
20 (-1)	100 (-1)	0.0301	1.81
70 (0)	250 (0)	0.0300	5.36
70 (0)	250 (0)	0.0303	5.26
70 (0)	250 (0)	0.0304	5.19
120 (1)	400 (1)	0.0303	6.79
20 (-1)	400 (1)	0.0304	3.63
120 (1)	100 (-1)	0.0302	2.88
20 (-1)	100 (-1)	0.0300	1.97
70 (0)	250 (0)	0.0302	5.09
70 (0)	250 (0)	0.0302	5.33
70 (0)	250 (0)	0.0303	5.30

Table S19. QS charcoal used as sunset yellow FCF removal treatment (numbers in parentheses correspond to the coded values used in the statistical analysis).

Time [min]	Sunset yellow FCF concentration [mg/L]	Charcoal mass [g]	mg/L Sunset yellow FCF captured/mg Charcoal
120 (1)	400 (1)	0.0304	2.74
20 (-1)	400 (1)	0.0304	2.17
120 (1)	100 (-1)	0.0301	0.70
20 (-1)	100 (-1)	0.0300	0.13
70 (0)	250 (0)	0.0300	1.98
70 (0)	250 (0)	0.0304	1.81
70 (0)	250 (0)	0.0302	1.72

Table S20. CQW charcoal used as sunset yellow FCF removal treatment (numbers in parentheses correspond to the coded values used in the statistical analysis).

Time [min]	Sunset yellow FCF concentration [mg/L]	Charcoal mass [g]	mg/L Sunset yellow FCF captured/mg Charcoal
120 (1)	400 (1)	0.0302	1.80
20 (-1)	400 (1)	0.0300	0.89
120 (1)	100 (-1)	0.0302	0.33
20 (-1)	100 (-1)	0.0302	0.01
70 (0)	250 (0)	0.0303	1.22
70 (0)	250 (0)	0.0300	1.18
70 (0)	250 (0)	0.0303	1.20

Table S21. QS-CO_2 used as sunset yellow FCF removal treatment (numbers in parentheses correspond to the coded values used in the statistical analysis).

Time [min]	Sunset yellow FCF concentration [mg/L]	Charcoal mass [g]	mg/L Sunset yellow FCF captured/mg Charcoal
120 (1)	400 (1)	0.0302	4.45
20 (-1)	400 (1)	0.0301	3.13
120 (1)	100 (-1)	0.0303	0.90
20 (-1)	100 (-1)	0.0304	0.41
70 (0)	250 (0)	0.0303	1.87
70 (0)	250 (0)	0.0303	1.82
70 (0)	250 (0)	0.0300	1.81

Table S22. CQW-CO_2 used as sunset yellow FCF removal treatment (numbers in parentheses correspond to the coded values used in the statistical analysis).

Time [min]	Sunset yellow FCF concentration [mg/L]	Charcoal mass [g]	mg/L Sunset yellow FCF captured/mg Charcoal
120 (1)	400 (1)	0.0302	6.56
20 (-1)	400 (1)	0.0300	6.66
120 (1)	100 (-1)	0.0300	2.02
20 (-1)	100 (-1)	0.0302	1.39
70 (0)	250 (0)	0.0301	2.75
70 (0)	250 (0)	0.0300	2.81
70 (0)	250 (0)	0.0302	2.59

Table S23. QS-H_2O used as sunset yellow FCF removal treatment (numbers in parentheses correspond to the coded values used in the statistical analysis).

Time [min]	Sunset yellow FCF concentration [mg/L]	Charcoal mass [g]	mg/L Sunset yellow FCF captured/mg Charcoal
120 (1)	400 (1)	0.0304	8.13
20 (-1)	400 (1)	0.0301	7.28
120 (1)	100 (-1)	0.0302	3.31
20 (-1)	100 (-1)	0.0300	3.27
70 (0)	250 (0)	0.0300	5.78
70 (0)	250 (0)	0.0301	5.78
70 (0)	250 (0)	0.0304	5.71

Table S24. CQW-H_2O used as sunset yellow FCF removal treatment (numbers in parentheses correspond to the coded values used in the statistical analysis).

Time [min]	Sunset yellow FCF concentration [mg/L]	Charcoal mass [g]	mg/L Sunset yellow FCF captured/mg Charcoal
120 (1)	400 (1)	0.0300	6.96
20 (-1)	400 (1)	0.0301	6.67
120 (1)	100 (-1)	0.0300	1.16
20 (-1)	100 (-1)	0.0300	0.83
70 (0)	250 (0)	0.0300	3.43
70 (0)	250 (0)	0.0302	3.35
70 (0)	250 (0)	0.0302	3.51

Table S25. QS-P used as sunset yellow FCF removal treatment (numbers in parentheses correspond to the coded values used in the statistical analysis).

Time [min]	Sunset yellow FCF concentration [mg/L]	Charcoal mass [g]	mg/L Sunset yellow FCF captured/mg Charcoal
120 (1)	400 (1)	0.0301	4.21
20 (-1)	400 (1)	0.0300	2.18
120 (1)	100 (-1)	0.0300	0.73
20 (-1)	100 (-1)	0.0303	0.21
70 (0)	250 (0)	0.0300	1.67
70 (0)	250 (0)	0.0304	1.59
70 (0)	250 (0)	0.0301	1.60

Table S26. CQW-P used as sunset yellow FCF removal treatment (numbers in parentheses correspond to the coded values used in the statistical analysis).

Time [min]	Sunset yellow FCF concentration [mg/L]	Charcoal mass [g]	mg/L Sunset yellow FCF captured/mg Charcoal
120 (1)	400 (1)	0.0300	7.21
20 (-1)	400 (1)	0.0304	6.34
120 (1)	100 (-1)	0.0300	1.21
20 (-1)	100 (-1)	0.0301	0.92
70 (0)	250 (0)	0.0302	2.42
70 (0)	250 (0)	0.0303	2.42
70 (0)	250 (0)	0.0301	2.45

Table S27. AC used as sunset yellow FCF removal treatment (numbers in parentheses correspond to the coded values used in the statistical analysis).

Time [min]	Sunset yellow FCF concentration [mg/L]	Charcoal mass [g]	mg/L Sunset yellow FCF captured/mg Charcoal
120 (1)	400 (1)	0.0303	10.14
20 (-1)	400 (1)	0.0304	7.25
120 (1)	100 (-1)	0.0303	3.29
20 (-1)	100 (-1)	0.0300	1.95
70 (0)	250 (0)	0.0300	6.45
70 (0)	250 (0)	0.0300	6.39
70 (0)	250 (0)	0.0301	6.39
120 (1)	400 (1)	0.0300	10.44
20 (-1)	400 (1)	0.0303	7.40
120 (1)	100 (-1)	0.0302	3.30
20 (-1)	100 (-1)	0.0300	2.18
70 (0)	250 (0)	0.0302	6.38
70 (0)	250 (0)	0.0302	6.42
70 (0)	250 (0)	0.0301	6.41

Figures:

TARTRAZINE

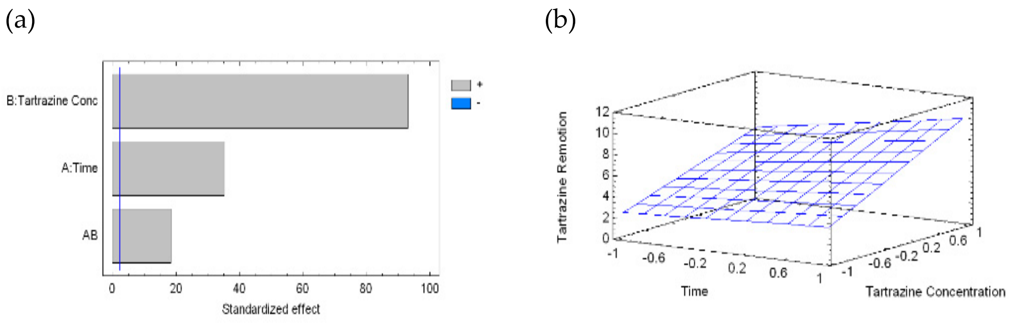

Figure S1. (a), Standardized Pareto chart for Tartrazine remotion by AC (Where: A, Time of interaction; B, Tartrazine concentration; AB, interaction. The line represents the critical t-value, 95% confidence); (b), Estimated response surface.

The regression equations of the models are:

$Tartrazine\ remotion\ by\ AC = 5.73429 + 1.04375 * \mathrm{A} + 2.75625 * \mathrm{B} + 0.54375 * \mathrm{AB}$

(R^2=93.04).

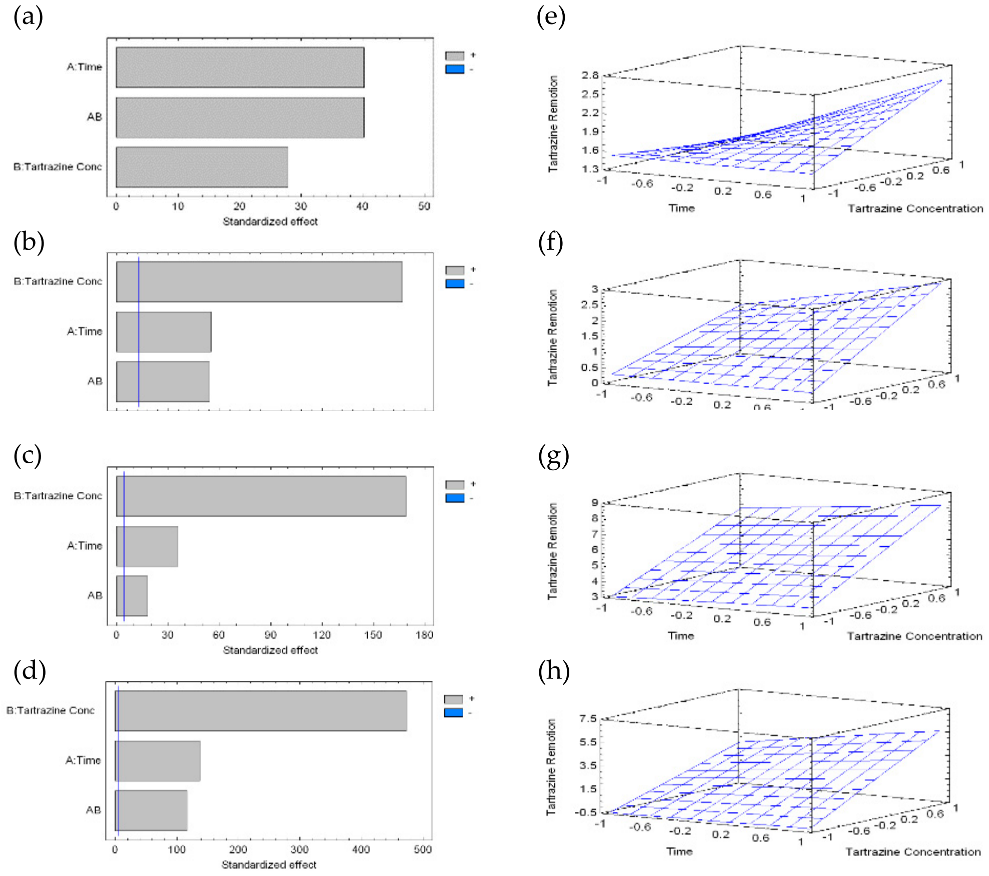

Figure S2. (a), (b), (c) and (d): Standardized Pareto charts for Tartrazine remotion by QS, QS-CO_2, QS-H_2O, and QS-P, respectively (Where: A, Time of interaction; B, Tartrazine concentration; AB, interaction. The line represents the critical t-value, 95% confidence); (e), (f), (g) and (h): Estimated response surfaces for Tartrazine capture by QS, QS-CO_2, QS-H_2O, and QS-P, respectively.

The regression equations of the models are:

$$Tartrazine\ remotion\ by\ QS = 1.73714 + 0.3075 * \mathrm{A} + 0.2125 * \mathrm{B} + 0.3075 * \mathrm{AB}$$

(R^2=45.15).

$$Tartrazine\ remotion\ by\ QS - CO_2 = 1.26429 + 0.3225 * \mathrm{A} + 0.9775 * \mathrm{B} + 0.3175 * \mathrm{AB}$$

(R^2=62.53).

$$Tartrazine\ remotion\ by\ QS - H_2O = 5.40286 + 0.45 * \mathrm{A} + 2.13 * \mathrm{B} + 0.225 * \mathrm{AB}$$

(R^2=96.25).

$$Tartrazine\ remotion\ by\ QS - P = 2.0 + 0.6875 * \mathrm{A} + 2.3675 * \mathrm{B} + 0.5825 * \mathrm{AB}$$

(R^2=43.35).

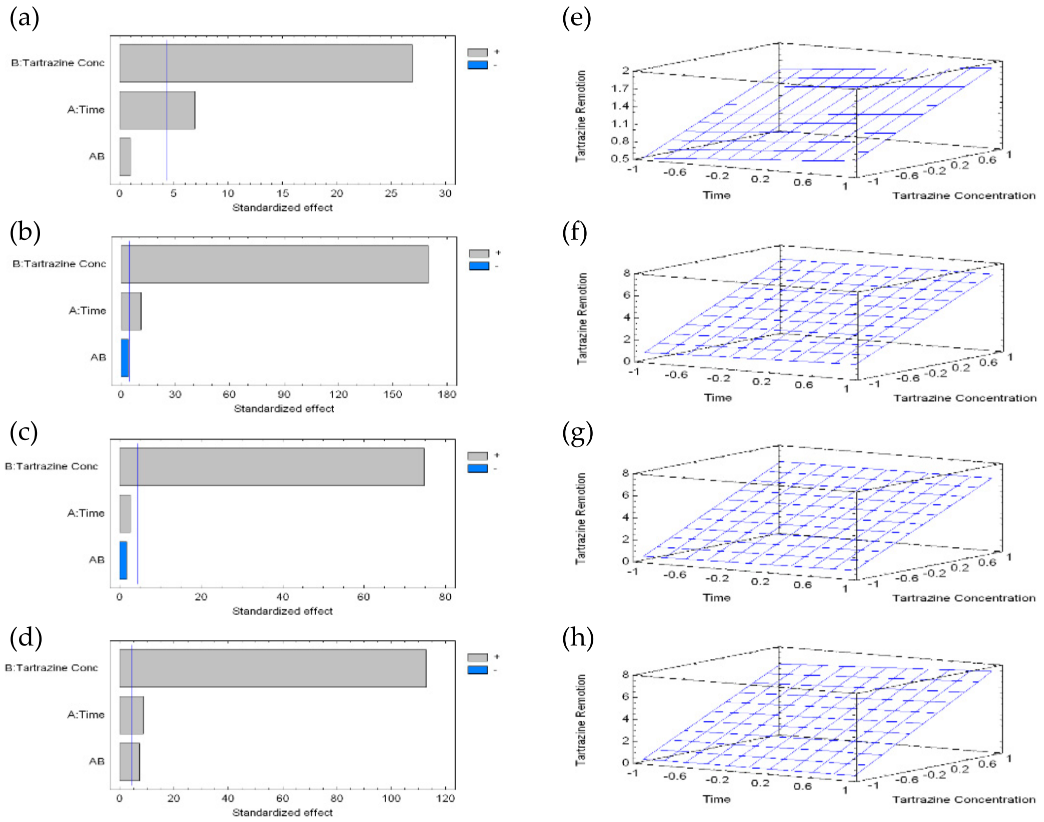

Figure S3. (a), (b), (c) and (d): Standardized Pareto charts for Tartrazine remotion by CQW, CQW-CO_2, CQW-H_2O, and CQW-P, respectively (Where: A, Time of interaction; B, Tartrazine concentration; AB, interaction. The line represents the critical t-value, 95% confidence); (e), (f), (g) and (h): Estimated response surfaces for Tartrazine capture by CQW, CQW-CO_2, CQW-H_2O, and CQW-P, respectively.

The regression equations of the models were:

$$Tartrazine\ remotion\ by\ CQW\ = 1.18286 + 0.14 * \mathrm{A} + 0.545 * \mathrm{B} + 0.02 * \mathrm{AB}$$

(R^2=39.23).

$$Tartrazine\ remotion\ by\ CQW - CO_2 = 4.01 + 0.1875 * \mathrm{A} + 2.9425 * \mathrm{B}$$

(R^2=88.69).

$$Tartrazine\ remotion\ by\ CQW - H_2O = 3.64571 + 3.0225 * \mathrm{B}$$

(R^2=85.29).

$$Tartrazine\ remotion\ by\ CQW - P = 3.69857 + 0.25 * \mathrm{A} + 3.31 * \mathrm{B} + 0.21 * \mathrm{A} * \mathrm{B}$$

(R^2=85.36).

CRYSTAL VIOLET

(a) (b)

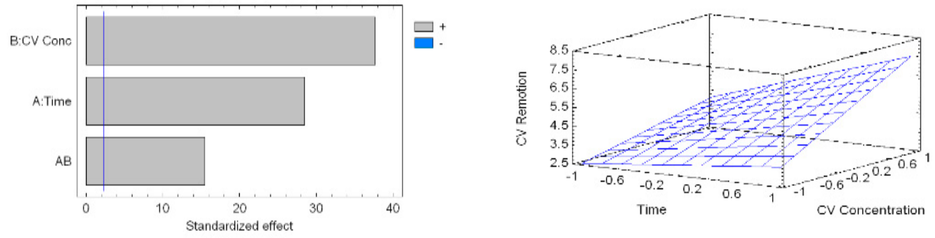

Figure S4. (a), Standardized Pareto chart for Crystal Violet remotion by AC (Where: A, Time of interaction; B, Crystal Violet concentration; AB, interaction. The line represents the critical t-value, 95% confidence); (b), Estimated response surface.

The regression equations of the models are:

$$Crystal\ Violet\ remotion\ by\ AC = 4.43429 + 1.08375 * \mathrm{A} + 1.43375 * \mathrm{B} + 0.58875 * \mathrm{AB}$$

(R^2=73.93).

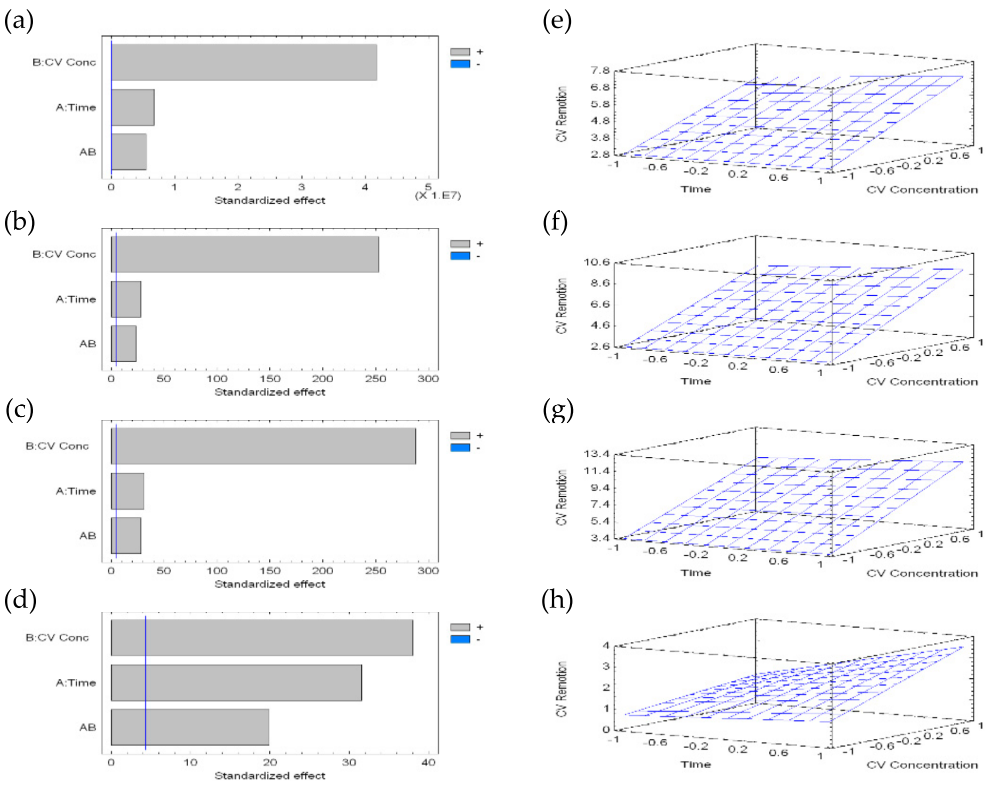

Figure S5. (a), (b), (c) and (d): Standardized Pareto charts for Crystal Violet remotion by QS, QS-CO_2, QS-H_2O, and QS-P, respectively (Where: A, Time of interaction; B, Crystal Violet concentration; AB, interaction. The line represents the critical t-value, 95% confidence); (e), (f), (g) and (h): Estimated response surfaces for Crystal Violet capture by QS, QS-CO_2, QS-H_2O, and QS-P, respectively.

The regression equations of the models are:

$$Crystal\ Violet\ remotion\ by\ QS = 4.67 + 0.285 * \mathrm{A} + 1.76 * \mathrm{B} + 0.23 * \mathrm{AB}$$

(R^2=78.82).

$$Crystal\ Violet\ remotion\ by\ QS - CO_2 = 5.58857 + 0.325 * \mathrm{A} + 2.915 * \mathrm{B} + 0.27 * \mathrm{A} * \mathrm{B}$$

(R^2=99.36).

$$Crystal\ Violet\ remotion\ by\ QS - H_2O = 7.07571 + 0.3925 * \mathrm{A} + 3.6225 * \mathrm{B} + 0.3525 * \mathrm{A} * \mathrm{B}$$

(R^2=98.12).

$$Crystal\ Violet\ remotion\ by\ QS - P = 1.72857 + 0.6375 * \mathrm{A} + 0.7675 * \mathrm{B} + 0.4025 * \mathrm{A} * \mathrm{B}$$

(R^2=46.34).

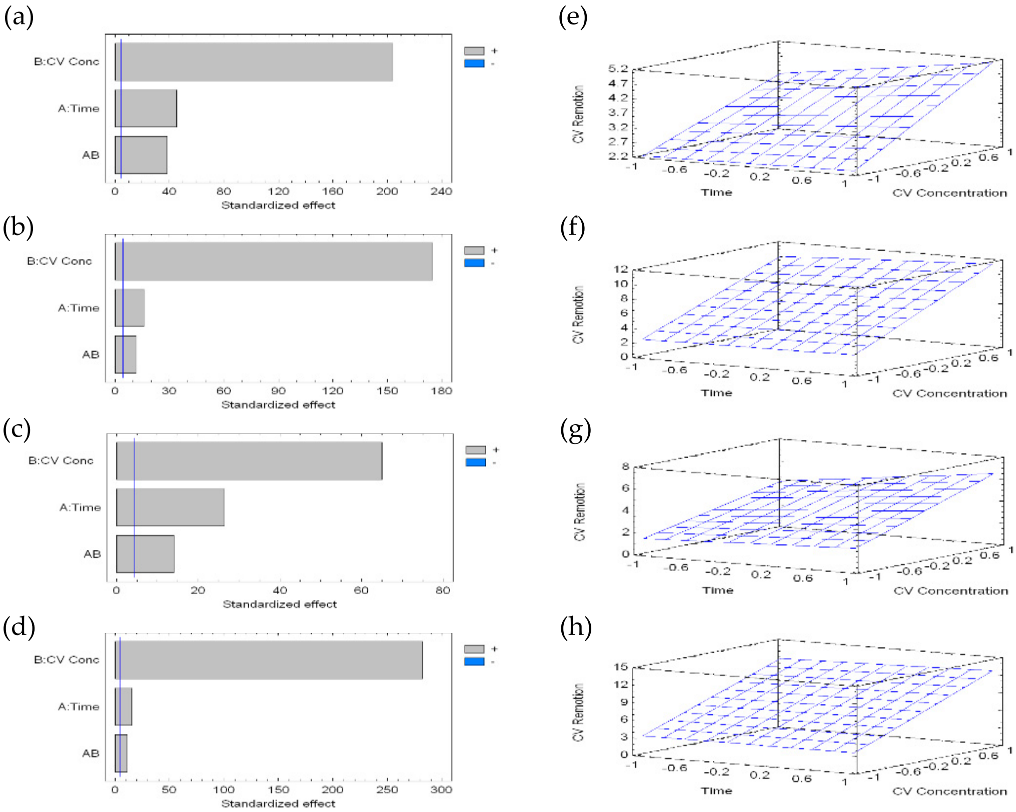

Figure S6. (a), (b), (c) and (d): Standardized Pareto charts for Crystal Violet remotion by CQW, CQW-CO_2, CQW-H_2O, and CQW-P, respectively (Where: A, Time of interaction; B, Crystal Violet concentration; AB, interaction. The line represents the critical t-value, 95% confidence); (e), (f), (g) and (h): Estimated response surfaces for Crystal Violet capture by CQW, CQW-CO_2, CQW-H_2O, and CQW-P, respectively.

The regression equations of the models are:

$$Crystal\ Violet\ remotion\ by\ CQW\ = 3.44857 + 0.2625 * \mathrm{A} + 1.1775 * \mathrm{B} + 0.2225 * \mathrm{AB}$$

(R^2=41.62).

$$Crystal\ Violet\ remotion\ by\ CQW - CO_2 = 6.72571 + 0.38 * \mathrm{A} + 4.135 * \mathrm{B} + 0.275 * \mathrm{A} * \mathrm{B}$$

(R^2=98.26).

$$Crystal\ Violet\ remotion\ by\ CQW - H_2O = 3.61714 + 0.7275 * \mathrm{A} + 1.7925 * \mathrm{B} + 0.3875 * \mathrm{A} * \mathrm{B}$$

(R^2=99.24).

$$Crystal\ Violet\ remotion\ by\ CQW - P = 7.88 + 0.2525 * \mathrm{A} + 4.5375 * \mathrm{B} + 0.1825 * \mathrm{A} * \mathrm{B}$$

(R^2=99.25).

SUNSET YELLOW FCF

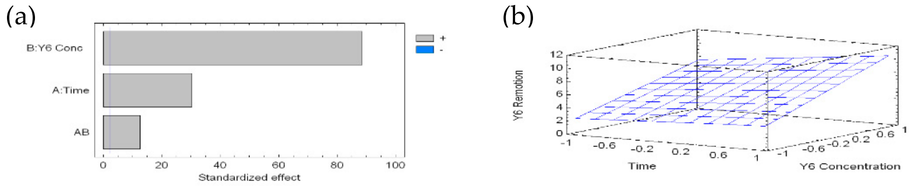

Figure S7. (a), Standardized Pareto chart for Sunset yellow FCF remotion by AC (Where: A, Time of interaction; B, Sunset yellow FCF concentration; AB, interaction. The line represents the critical t-value, 95% confidence); (b), Estimated response surface.

The regression equations of the models are:

$Sunset\ yellow\ FCF\ \ remotion\ by\ AC = 6.02786 + 1.04875 * \mathrm{A} + 3.06375 * \mathrm{B} + 0.43375 * \mathrm{AB}$

(R^2=97.62).

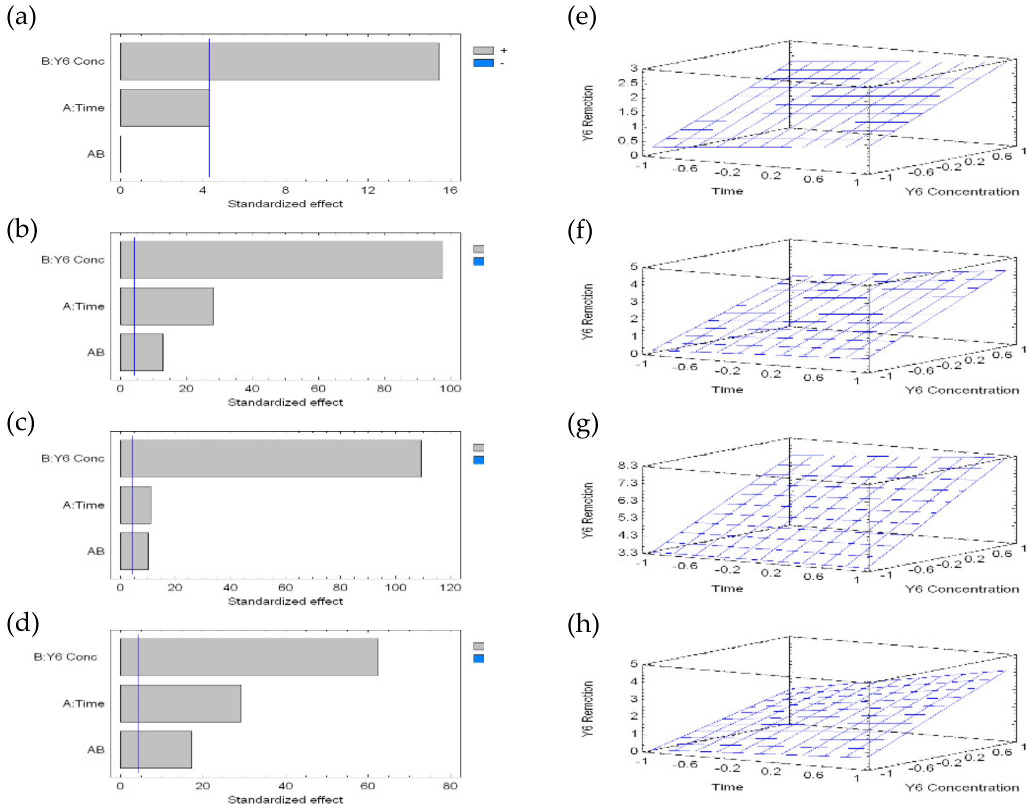

Figure S8. (a), (b), (c) and (d): Standardized Pareto charts for Sunset yellow FCF remotion by QS, QS-CO_2, QS-H_2O, and QS-P, respectively (Where: A, Time of interaction; B, Sunset yellow FCF concentration; AB, interaction. The line represents the critical t-value, 95% confidence); (e), (f), (g) and (h): Estimated response surfaces for Sunset yellow FCF capture by QS, QS-CO_2, QS-H_2O, and QS-P, respectively.

The regression equations of the models are:

$$Sunset\ yellow\ FCF\ remotion\ by\ QS = 1.60714 + 0.285 * \mathrm{A} + 1.02 * \mathrm{B}$$

(R^2=87.02).

$$Sunset\ yellow\ FCF\ \ remotion\ by\ QS - CO_2 = 2.05571 + 0.4525 * \mathrm{A} + 1.5675 * \mathrm{B} + 0.2075 * \mathrm{A} * \mathrm{B}$$

(R^2=95.28).

$$Sunset\ yellow\ FCF\ \ remotion\ by\ QS{-}H_2O = 5.60857 + 0.2225 * \mathrm{A} + 2.2075 * \mathrm{B} + 0.2025 * \mathrm{A} * \mathrm{B}$$

(R^2=98.81).

$$Sunset\ yellow\ FCF\ \ remotion\ by\ QS - P = 1.74143 + 0.6375 * \mathrm{A} + 1.3625 * \mathrm{B} + 0.3775 * \mathrm{A} * \mathrm{B}$$

(R^2=98.33).

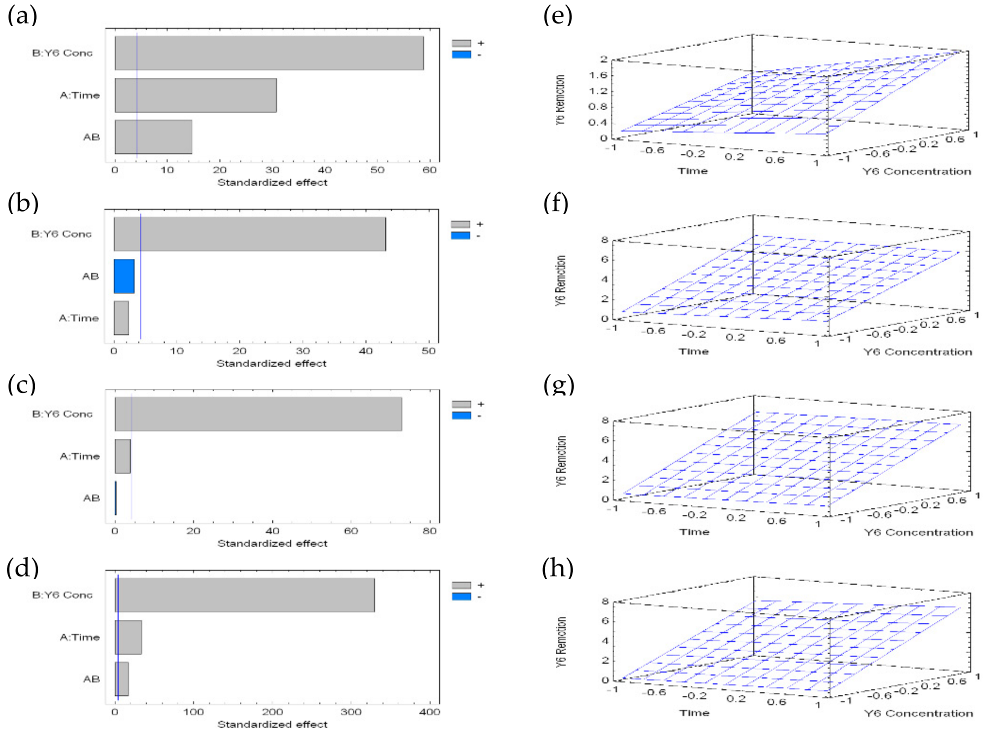

Figure S9. (a), (b), (c) and (d): Standardized Pareto charts for Sunset yellow FCF remotion by CQW, CQW-CO_2, CQW-H_2O, and CQW-P, respectively (Where: A, Time of interaction; B, Sunset yellow FCF concentration; AB, interaction. The line represents the critical t-value, 95% confidence); (e), (f), (g) and (h): Estimated response surfaces for Sunset yellow FCF capture by CQW, CQW-CO_2, CQW-H_2O, and CQW-P, respectively.

The regression equations of the models are:

$$Sunset\ yellow\ FCF\ \ remotion\ by\ CQW = 0.947143 + 0.3075 * \mathrm{A} + 0.5875 * \mathrm{B} + 0.1475 * \mathrm{AB}$$

(R^2=69.16).

$$Sunset\ yellow\ FCF\ remotion\ by\ CQW - CO_2 = 3.54 + 2.4525 * \mathrm{B}$$

(R^2=74.25).

$$Sunset\ yellow\ FCF\ remotion\ by\ CQW - H_2O = 3.70143 + 2.91 * \mathrm{B}$$

(R^2=97.68).

$$Sunset\ yellow\ FCF\ remotion\ by\ CQW - P = 3.28143 + 0.29 * \mathrm{A} + 2.855 * \mathrm{B} + 0.145 * \mathrm{A} * \mathrm{B}$$

(R^2=79.33).

The optimum experimental conditions for the capture of dyes by all samples are as follows:

Interaction time of 120 min, and dye concentration of 400 mg/L.

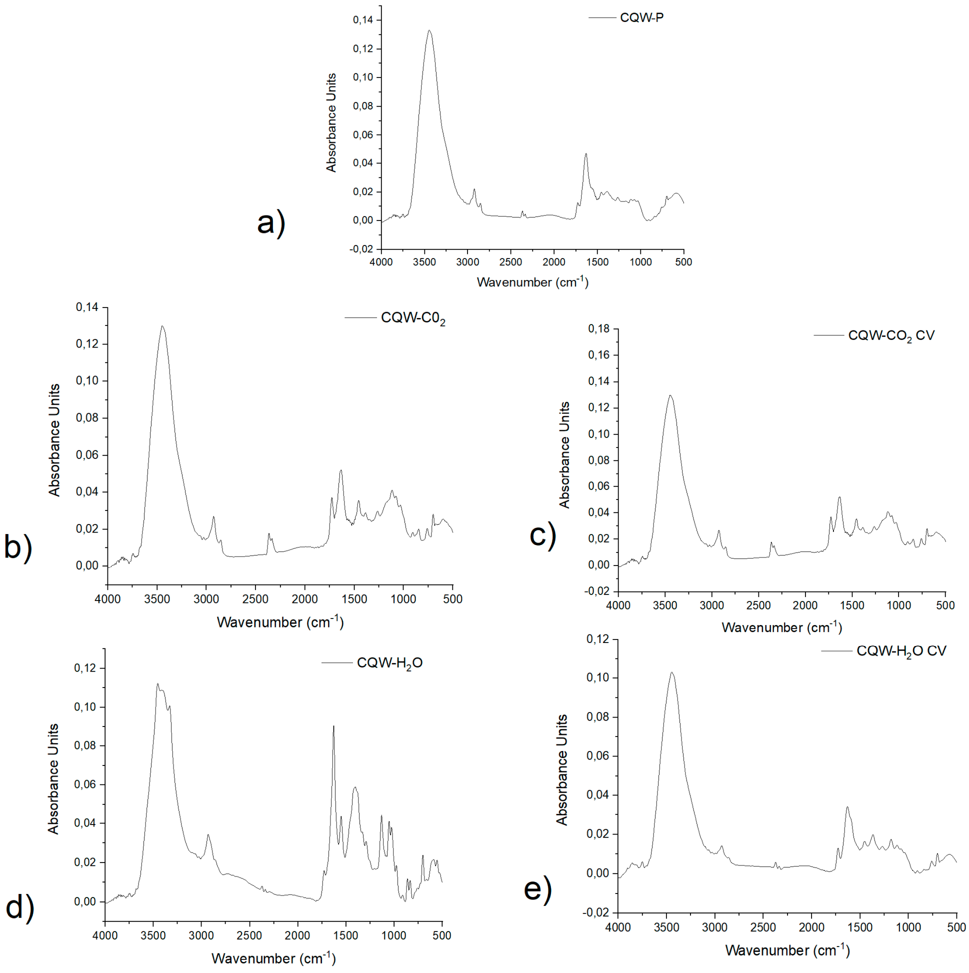

Figure S10. FT-IR spectra of CQW-P (a), CQW-CO_2 (b), CQW-CO_2 CV (c), CQW-H_2O (d), and CQW-H_2O CV (e).

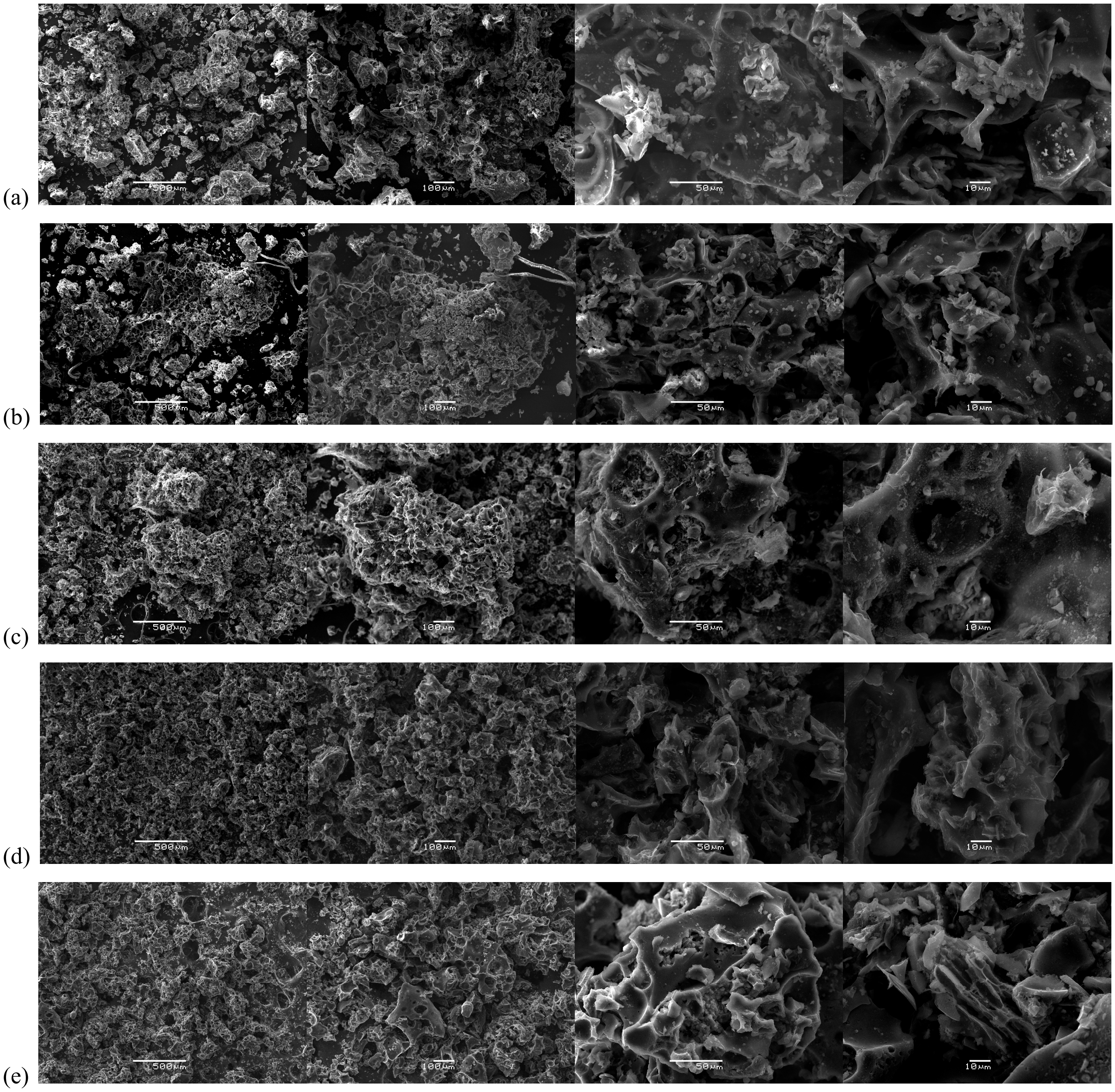

Figure S11. SEM images of the CQW-P (a), CQW-CO_2 (b), CQW-CO_2 CV (c), CQW-H_2O (d), and CQW-H_2O CV (e). All images were taken with magnification 500×, 100×, 50× and 10×, respectively.
